# Early treatment of COVID-19 with anakinra guided by soluble urokinase plasminogen receptor plasma levels: a double-blind, randomized controlled phase 3 trial

**DOI:** 10.1038/s41591-021-01499-z

**Published:** 2021-09-03

**Authors:** Evdoxia Kyriazopoulou, Garyfallia Poulakou, Haralampos Milionis, Simeon Metallidis, Georgios Adamis, Konstantinos Tsiakos, Archontoula Fragkou, Aggeliki Rapti, Christina Damoulari, Massimo Fantoni, Ioannis Kalomenidis, Georgios Chrysos, Andrea Angheben, Ilias Kainis, Zoi Alexiou, Francesco Castelli, Francesco Saverio Serino, Maria Tsilika, Petros Bakakos, Emanuele Nicastri, Vassiliki Tzavara, Evangelos Kostis, Lorenzo Dagna, Panagiotis Koufargyris, Katerina Dimakou, Spyridon Savvanis, Glykeria Tzatzagou, Maria Chini, Giulio Cavalli, Matteo Bassetti, Konstantina Katrini, Vasileios Kotsis, George Tsoukalas, Carlo Selmi, Ioannis Bliziotis, Michael Samarkos, Michael Doumas, Sofia Ktena, Aikaterini Masgala, Ilias Papanikolaou, Maria Kosmidou, Dimitra-Melia Myrodia, Aikaterini Argyraki, Chiara Simona Cardellino, Katerina Koliakou, Eleni-Ioanna Katsigianni, Vassiliki Rapti, Efthymia Giannitsioti, Antonella Cingolani, Styliani Micha, Karolina Akinosoglou, Orestis Liatsis-Douvitsas, Styliani Symbardi, Nikolaos Gatselis, Maria Mouktaroudi, Giuseppe Ippolito, Eleni Florou, Antigone Kotsaki, Mihai G. Netea, Jesper Eugen-Olsen, Miltiades Kyprianou, Periklis Panagopoulos, George N. Dalekos, Evangelos J. Giamarellos-Bourboulis

**Affiliations:** 1 4th Department of Internal Medicine, National and Kapodistrian University of Athens, Medical School, Athens, Greece; 2 3rd Department of Internal Medicine, National and Kapodistrian University of Athens, Medical School, Athens, Greece; 3 1st Department of Internal Medicine, University of Ioannina, Medical School, Ioannina, Greece; 4 1st Department of Internal Medicine, Aristotle University of Thessaloniki, Medical School, Thessaloniki, Greece; 5 1st Department of Internal Medicine, G. Gennimatas General Hospital of Athens, Athens, Greece; 6 2nd Department of Pulmonary Medicine, Sotiria General Hospital of Chest Diseases, Athens, Greece; 7 Department of Internal Medicine, Elpis General Hospital, Athens, Greece; 8 Dipartimento Scienze di Laboratorio e Infettivologiche - Fondazione Policlinico Gemelli IRCCS, Roma, Italy; 9 1st Department of Critical Care and Pulmonary Medicine, Medical School, National and Kapodistrian University of Athens, Evangelismos General Hospital, Athens, Greece; 10 2nd Department of Internal Medicine, Tzaneio General Hospital of Piraeus, Athens, Greece; 11 Department of Infectious Tropical Diseases and Microbiology, IRCSS Sacro Cuore Hospital, Negrar, Verona, Italy; 12 10th Department of Pulmonary Medicine, Sotiria General Hospital of Chest Diseases of Athens, Athens, Greece; 13 2nd Department of Internal Medicine, Thriasio General Hospital of Eleusis, Athens, Greece; 14 Spedali Civili, Brescia ASST Spedali Civili Hospital, University of Brescia, Brescia, Italy; 15 Department of Internal Medicine, Hospital of Jesolo, Jesolo, Italy; 16 1st Department of Chest Medicine, National and Kapodistrian University of Athens, Medical School, Athens, Greece; 17 Department of Internal Medicine, Spallanzani Institute of Rome, Rome, Italy; 18 1st Department of Internal Medicine, Korgialeneion-Benakeion General Hospital, Athens, Greece; 19 Department of Therapeutics, National and Kapodistrian University of Athens, Medical School, Athens, Greece; 20 Unit of Immunology, Rheumatology, Allergy and Rare Diseases (UnIRAR), IRCCS Ospedale San Raffaele & Vita-Salute San Raffaele University, Milan, Italy; 21 5th Department of Pulmonary Medicine, Sotiria General Hospital of Chest Diseases, Athens, Greece; 22 1st Department of Internal Medicine, Papageorgiou General Hospital of Thessaloniki, Thessaloniki, Greece; 23 3rd Department of Internal Medicine and Infectious Diseases Unit, Korgialeneion-Benakeion General Hospital, Athens, Greece; 24 Infectious Diseases Clinic, Ospedale Policlinico San Martino IRCCS and Department of Health Sciences, University of Genova, Genova, Italy; 25 3rd Department of Internal Medicine, Aristotle University of Thessaloniki, Medical School, Thessaloniki, Greece; 26 4th Department of Pulmonary Medicine, Sotiria General Hospital of Chest Diseases, Athens, Greece; 27 Department of Biomedical Sciences, Humanitas University, Milan, Italy & IRCCS Humanitas Research Hospital, Milan, Italy; 28 1st Department of Internal Medicine, Asklepieio General Hospital of Voula, Athens, Greece; 29 1st Department of Internal Medicine, National and Kapodistrian University of Athens, Medical School, Athens, Greece; 30 2nd Department of Propedeutic Medicine, Aristotle University of Thessaloniki, Medical School, Thessaloniki, Greece; 31 2nd Department of Internal Medicine, Konstantopouleio General Hospital, Athens, Greece; 32 Department of Pulmonary Medicine, General Hospital of Kerkyra, Corfu, Greece; 33 Department of Internal Medicine, Sotiria General Hospital of Chest Diseases, Athens, Greece; 34 Hellenic Institute for the Study of Sepsis, Athens, Greece; 35 Department of Internal Medicine, University of Patras, Rion, Greece; 36 1st Department of Internal Medicine, Thriasio General Hospital of Eleusis, Athens, Greece; 37 Department of Medicine and Research Laboratory of Internal Medicine, National Expertise Center of Greece in Autoimmune Liver Diseases, General University Hospital of Larissa, Larissa, Greece; 38 Department of Internal Medicine and Center for Infectious Diseases, Radboud University, Nijmegen, The Netherlands; 39 Department of Immunology and Metabolism, Life and Medical Sciences Institute, University of Bonn, Bonn, Germany; 40 Department of Clinical Research, Copenhagen University Hospital, Amager and Hvidovre, Denmark; 41 2nd Department of Internal Medicine, Democritus University of Thrace, Medical School, Alexandroupolis, Greece

**Keywords:** Medical research, Randomized controlled trials

## Abstract

Early increase of soluble urokinase plasminogen activator receptor (suPAR) serum levels is indicative of increased risk of progression of coronavirus disease 2019 (COVID-19) to respiratory failure. The SAVE-MORE double-blind, randomized controlled trial evaluated the efficacy and safety of anakinra, an IL-1α/β inhibitor, in 594 patients with COVID-19 at risk of progressing to respiratory failure as identified by plasma suPAR ≥6 ng ml^−1^, 85.9% (*n* = 510) of whom were receiving dexamethasone. At day 28, the adjusted proportional odds of having a worse clinical status (assessed by the 11-point World Health Organization Clinical Progression Scale (WHO-CPS)) with anakinra, as compared to placebo, was 0.36 (95% confidence interval 0.26–0.50). The median WHO-CPS decrease on day 28 from baseline in the placebo and anakinra groups was 3 and 4 points, respectively (odds ratio (OR) = 0.40, *P* < 0.0001); the respective median decrease of Sequential Organ Failure Assessment (SOFA) score on day 7 from baseline was 0 and 1 points (OR = 0.63, *P* = 0.004). Twenty-eight-day mortality decreased (hazard ratio = 0.45, *P* = 0.045), and hospital stay was shorter.

## Abstract (web summary)

The SAVE-MORE phase 3 study demonstrates the efficacy of anakinra, an IL-1α/β inhibitor, in patients with COVID-19 and high serum levels of soluble plasminogen activator receptor.

## Main

COVID-19 can have an unpredictable clinical course. Patients might suddenly deteriorate into severe respiratory failure, defined as a respiratory ratio (partial oxygen pressure (PaO_2_)/fraction of inspired oxygen (FIO_2_)) below 150 mmHg, necessitating non-invasive ventilation (NIV) or mechanical ventilation (MV). Early recognition of patients at risk of progressing to severe disease and timely onset of targeted treatment are of utmost importance.

Research by our group and others has shown that suPAR is a biomarker that predicts progression to severe respiratory failure or death in patients with COVID-19. suPAR is increased earlier in disease progression than other biomarkers, including C-reactive protein (CRP), interleukin (IL)-6, ferritin and D-dimers^1,2^. suPAR denotes the presence of danger-associated molecular patterns (DAMPs), namely calprotectin (S100A8/A9) and IL-1α^3,4^, both of which contribute to pathogenic inflammation in COVID-19. Calprotectin stimulated aberrant production of IL-1β by circulating monocytes^4^, and inhibition of IL-1α prevented the development of strong pro-inflammatory responses in a COVID-19-like animal model^3^. Early elevations in suPAR signify risk of progression to severe respiratory failure or death and also refractoriness to full recovery^1,2^. These observations led us to propose a two-step strategy for managing patients with COVID-19, which used elevated suPAR levels to first identify patients at risk of progressing to severe respiratory failure or death and then to initiate early targeted treatment with anakinra, a recombinant IL-1 receptor antagonist that blocks the activity of both IL-1α and IL-1β. The open-label, phase 2 SAVE study was conducted as a proof of concept for this approach^5^. Results showed a 70% decrease in the relative risk of progression to severe respiratory failure and a significant reduction in 28-d mortality with anakinra treatment compared to standard of care.

The SAVE-MORE study (suPAR-guided Anakinra treatment for Validation of the risk and Early Management Of seveRE respiratory failure by COVID-19) is a pivotal, confirmatory, phase 3, double-blind randomized controlled trial that evaluated the efficacy and safety of early initiation of anakinra treatment in hospitalized patients with moderate or severe COVID-19. The primary objective was to evaluate the efficacy and safety of early anakinra administration on the 11-point ordinal WHO-CPS on day 28 from the start of treatment.

## Results

### Patients

**Fig. 1:**
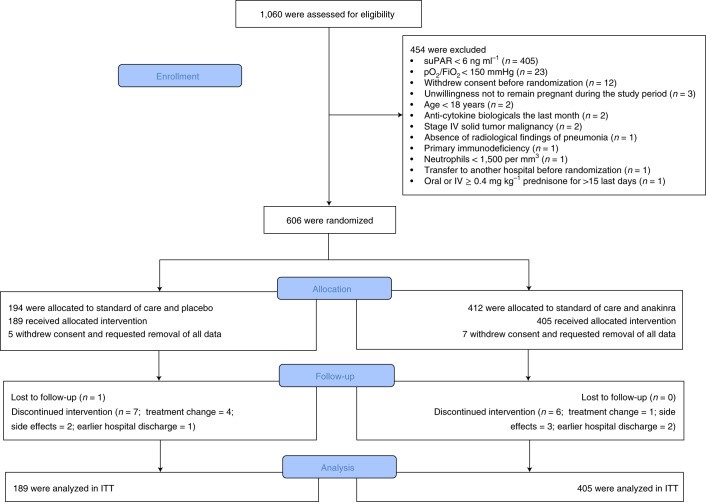
Study flow chart. IV, intravenous. Source data

From 23 December 2020 to 31 March 2021, 1,060 patients with molecular definition of COVID-19 by positive polymerase chain reaction (PCR) for severe acute respiratory syndrome coronavirus 2 (SARS-CoV-2) were screened, and 606 were randomized at 37 study sites (Methods). The main reason for exclusion from the study was suPAR <6 ng ml^−1^. Twelve patients withdrew consent and requested removal of all data, leaving a final intention-to-treat (ITT) analysis cohort of 594 patients. All patients received standard of care; 189 patients were allocated to the placebo arm; and 405 patients were allocated to the anakinra arm. Only one patient was lost to follow-up (Fig. 1). Baseline characteristics and co-administered treatments were similar between the two treatment arms (Table 1). Overall, 91.6% of patients had severe pneumonia as defined by the WHO classification for COVID-19. Patients not on dexamethasone at baseline started dexamethasone in compliance with standard of care after the start of the study drug.

**Table 1:** Baseline characteristics of patients

	Placebo (*n* = 189)	Anakinra (*n* = 405)	All patients (*n* = 594)
Age, years, mean (s.d.)	61.5 (11.3)	62.0 (11.4)	61.9 (12.1)
Male sex, *n* (%)	108 (57.1)	236 (58.3)	344 (57.9)
Mean BMI (s.d.)	29.8 (5.6)	29.4 (5.5)	29.5 (5.5)
Charlson’s comorbidity index, mean (s.d.)	2.2 (1.5)	2.3 (1.6)	2.2 (1.6)
SOFA score, mean (s.d.)	2.5 (1.2)	2.4 (1.1)	2.4 (1.1)
WHO classification for COVID-19 at the time of screening, (%)			
Moderate pneumonia	27 (14.3)	82 (20.2)	109 (18.4)
Severe pneumonia^a^	162 (85.7)	323 (79.8)	485 (81.6)
WHO classification for COVID-19 before start of the study drug, (%)			
Moderate pneumonia	11 (5.8)	39 (9.6)	50 (8.4)
Severe pneumonia^a^	178 (94.2)	366 (90.4)	544 (91.6)
Days to start of the study drug, median (Q1–Q3)			
From symptom onset	9 (7–11)	9 (7–12)	9 (7–11)
From hospital admission	2 (2–3)	2 (2–3)	2 (2–3)
Laboratory values before start of the study drug, median (Q1–Q3)			
White blood cell count, cells per mm^3^	5,910 (4,280–8,300)	5,980 (4,320–8,180)	5,950 (4,310–8,200)
Lymphocyte count, cells per mm^3^	730 (560–1,090)	815 (570–1,110)	800 (565–1,100)
CRP, mg L^−1^	51.4 (25.2–98.5)	50.5 (25.2–100.2)	50.6 (25.3–99.7)
IL-6, pg ml^−1^	20.1 (7.4–45.0)	15.5 (6.7–39.3)	16.8 (7.0–39.8)
Ferritin, ng ml^−1^	628.6 (293.5–1,062.3)	558.9 (294.1–1,047.0)	585.2 (294.5–1,047.0)
Serum soluble uPAR, ng ml^−1^	7.5 (6.9–9.3)	7.6 (7.0–9.1)	7.6 (6.9–9.1)
PaO_2_/FiO_2_	223 (168–297)	239 (186–302)	237 (181–301)
Comorbidities, *n* (%)			
Type 2 diabetes mellitus	28 (14.8)	66 (16.3)	94 (15.8)
Chronic heart failure	5 (2.6)	13 (3.2)	18 (3.0)
Chronic renal disease	1 (0.5)	9 (2.2)	10 (1.7)
Chronic obstructive pulmonary disease	9 (4.8)	15 (3.7)	24 (4.0)
Coronary heart disease	13 (6.9)	28 (6.9)	41 (6.9)
Atrial fibrillation	8 (4.2)	20 (4.9)	28 (4.7)
Depression	9 (4.8)	25 (6.2)	34 (5.7)
Administered doses of study drug, mean (s.d.)	8.7 (2.0)	8.4 (2.1)	8.6 (1.8)
Oxygen administration, *n* (%)	178 (94.2)	366 (90.4)	544 (91.6)
Co-administered medications, *n* (%)			
Remdesivir	141 (74.6)	298 (73.6)	439 (73.9)
Dexamethasone at enrollment	168 (88.9)	342 (84.4)	510 (85.9)
Low-molecular-weight heparin	175 (92.6)	385 (95.1)	560 (94.3)
β-lactamase inhibitors	10 (5.3)	23 (5.7)	33 (5.6)
Piperacillin/tazobactam	36 (19.0)	64 (15.8)	100 (16.8)
Ceftriaxone	85 (45.0)	155 (38.3)	240 (40.4)
Ceftaroline	32 (16.9)	75 (18.5)	107 (18.0)
Respiratory fluoroquinolone	24 (12.7)	53 (13.1)	77 (13.0)
Azithromycin	35 (18.5)	76 (18.8)	111 (18.7)
Any glycopeptide	19 (10.1)	24 (5.9)	43 (7.2)
Linezolid	22 (11.6)	45 (11.1)	67 (11.3)

^a^Defined as oxygen saturation less than 90% or more than 30 breaths per minute or signs of respiratory distress

Q, quartile.

### Primary and secondary endpoints

**Fig. 2:**
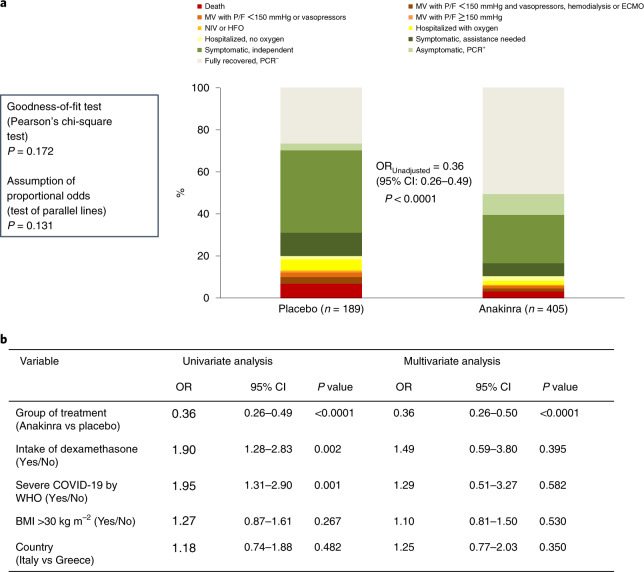
Study primary endpoint. **a**, Distribution of the WHO-CPS scores at day 28 of patients allocated to treatment with placebo and to treatment with anakinra. Comparison is done by unadjusted ordinal regression analysis; the ORs of the 95% CIs are provided. The exact *P* value of the unadjusted ordinal regression analysis is 3.6 × 10^−10^. The two tests of the assumptions of the ordinal regression analysis are also provided. **b**, Univariate and multivariate ordinal regression analysis of the WHO-CPS scores at day 28. The exact *P* value of the effect of anakinra versus placebo treatment in the multivariate analysis is 7.7 × 10^−10^. Covariates entered in the multivariate model were those used for stratified randomization according to advice received from the COVID-ETF. ECMO, extracorporeal membrane oxygenation; P/F, respiratory ratio. Source data

The distributions of patient scores on the 11-point WHO-CPS in the two treatment arms at day 28 (primary outcome) are shown in Table 2 and Fig. 2a. In brief, 50.4% (204/405) of patients receiving anakinra had fully recovered with no viral RNA detected on day 28 compared to 26.5% (50/189) of patients receiving placebo, and 3.2% (13/405) and 6.9% (13/189) of patients in the anakinra and placebo arms, respectively, died. Overall, the unadjusted proportional odds of having a worse score on the 11-point WHO-CPS at day 28 with anakinra was 0.36 versus placebo (95% confidence interval (CI) 0.26–0.49, *P* < 0.0001; ordinal regression analysis) (Fig. 2a). The assumptions of the ordinal regression analysis—that is, the goodness-of-fit test and the parallel lines test—were not statistically significant, indicating that there was a high likelihood of the treatment effect size being homogeneous for all 11 points of the WHO-CPS.

**Table 2:** Primary and secondary study endpoints

	Placebo (*n* = 189)	Anakinra (*n* = 405)	OR (95% CI)	*P* value
WHO-CPS by day 28			0.36 (0.26–0.50)	<0.0001
Fully recovered PCR^−^, *n* (%)	50 (26.5)	204 (50.4)		
Asymptomatic PCR^+^, *n* (%)	6 (3.2)	40 (9.9)		
Symptomatic independent, *n* (%)	74 (39.2)	93 (23.0)		
Symptomatic assistance needed, *n* (%)	21 (11.1)	25 (6.2)		
Hospitalized with no need for oxygen, *n* (%)	3 (1.6)	9 (2.2)		
Hospitalized with nasal/mask oxygen, *n* (%)	10 (5.3)	8 (2.0)		
Need for HFO or NIV, *n* (%)	1 (0.5)	1 (0.2)		
MV with P/F > 150 mmHg, *n* (%)	1 (0.5)	1 (0.2)		
MV with P/F < 150 mmHg or vasopressors, *n* (%)	4 (2.1)	5 (1.2)		
MV with P/F < 150 mmHg and vasopressors or hemodialysis or ECMO, *n* (%)	6 (3.2)	6 (1.5)		
Dead, *n* (%)	13 (6.9)	13 (3.2)		
Absolute decrease of WHO-CPS at day 28 from baseline day 1, median (IQR)	3 (2.5)	4 (2.0)	0.40 (0.29–0.55)	<0.0001
Absolute decrease of WHO-CPS at day 14 from baseline day 1, median (IQR)	2 (3.0)	3 (2.0)	0.63 (0.46–0.86)	0.003
Absolute decrease of SOFA score at day 7 from baseline day 1, median (IQR)	0 (1)	1 (2)	0.64 (0.47–0.88)	0.007
Median (IQR) time to hospital discharge, d	12 (8.5)	11 (7.8)	1.22 (1.02–1.47)^a^	0.033
Median (IQR) time of ICU stay, d^b^	14 (22)	10 (21)	2.33 (1.11–4.92)^a^	0.026

The comparisons between the two groups for the primary endpoint and for the secondary endpoints (absolute decrease of WHO-CPS at day 28 from baseline day 1, absolute decrease of WHO-CPS at day 14 from baseline day 1 and absolute decrease of SOFA score at day 7 from baseline day 1) were performed by multivariate ordinal regression analyses. The time to hospital discharge and the time to ICU discharge were compared by univariate Cox regression analysis. The exact *P* value for the comparison of WHO-CPS scores by day 28 is 7.7 × 10^−^^10^, and the exact *P* value for the absolute decrease of WHO-CPS score at day 28 from baseline day 1 is 1.4 × 10^−8^.

^a^Hazard ratio

^b^Only for patients admitted in the ICU

ECMO, extra corporeal membrane oxygenation; IQR, interquartile range; P/F, respiratory ratio.

Multivariate ordinal regression analysis of the primary outcome was performed using the stratification factors for randomization as independent variables—that is, disease severity, intake of dexamethasone, body mass index (BMI) >30 kg m^−^^2^ and country (Fig. 2b). Results of the univariate analysis showed that dexamethasone treatment and baseline severe COVID-19 were associated with higher odds, and anakinra treatment with lower odds, of a worse (higher) WHO-CPS score at day 28. Of note, the observed higher odds of a worse outcome with dexamethasone likely reflects the administration of dexamethasone to patients with severe pneumonia rather than a true detrimental effect of dexamethasone. In the multivariate analysis, treatment with anakinra was the only independent variable associated with the primary outcome. Compared to placebo, the adjusted proportional odds of having a worse score on the 11-point WHO-CPS at day 28 with anakinra was 0.36 (95% CI 0.26–0.50, *P* < 0.0001) (Fig. 2b).

**Extended Data Fig. 1:**
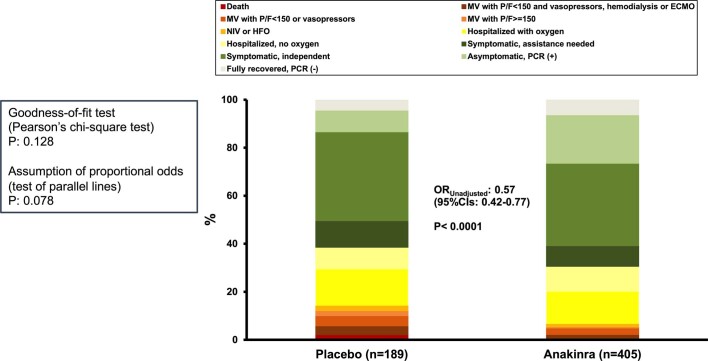
First supporting analysis of the study primary endpoint. Distribution of the World Health Organization (WHO) Clinical Progression Scale (CPS) at day 14 of patients allocated to treatment with placebo and to treatment with anakinra. Comparison is done by the unadjusted ordinal regression analysis; the odds ratio (OR) and the 95% confidence intervals (CIs) are provided. The exact P value is 3.9 ×10-4. The two tests of the assumptions of the ordinal regression analysis are also provided. Abbreviations CI: confidence interval; ECMO: extracorporeal membrane oxygenation; HFO: high flow oxygen; MV: mechanical ventilation; NIV: non-invasive ventilation; OR: odds ratio; PCR: polymerase chain reaction; P/F: respiratory ratio. Source data

**Extended Data Fig. 2:**
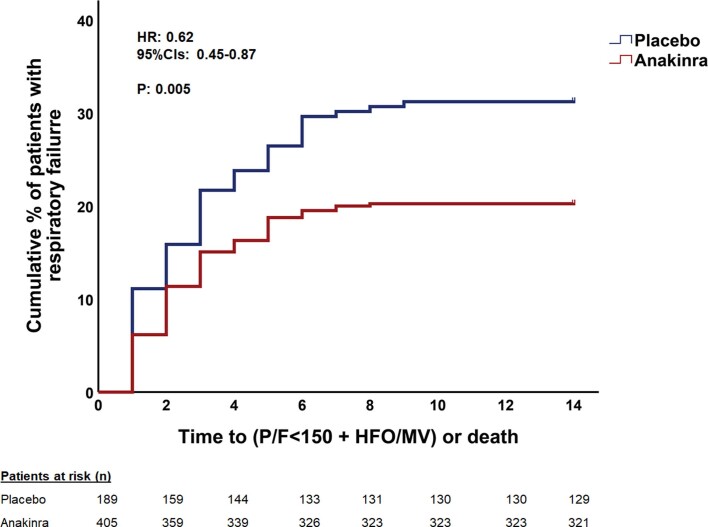
Time to progression into severe respiratory failure. Severe respiratory failure is defined as respiratory ratio-PF < 150 necessitating high flow oxygen/non-invasive ventilation/mechanical ventilation or death) by day 14. Comparison is done by univariate Cox regression analysis. The hazard ratio (HR) and the 95% confidence intervals (CIs) are provided. Abbreviations HFO: high flow oxygen; MV: mechanical ventilation; n: number; P/F: respiratory ratio; SoC; standard-of-care. Source data

**Extended Data Fig. 3:**
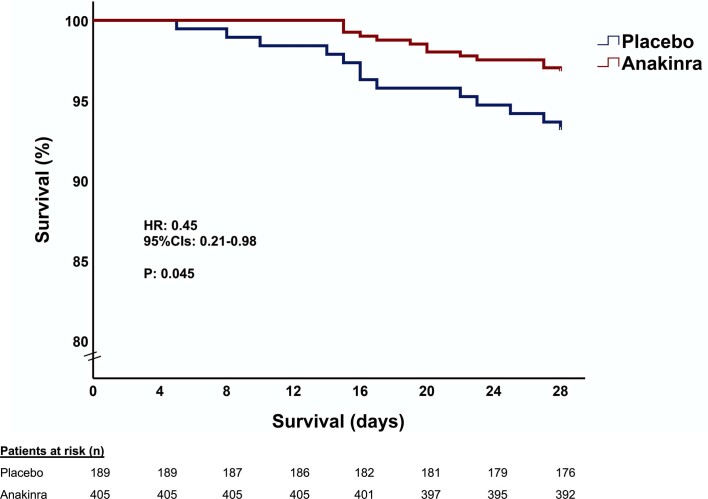
Survival analysis of enrolled patients. Comparison is done by univariate Cox regression analysis. Abbreviations: CI: confidence intervals; HR: hazard ratio; n: number. Source data

Three pre-specified confirmatory analyses of the primary endpoint were performed: (1) comparison of patients’ WHO-CPS score distributions in the anakinra versus placebo arms at day 14; (2) analysis of the proportion of patients with persistent disease at day 28 in each treatment arm (no disease persistence defined as full recovery with no viral RNA detected, WHO-CPS score of 0; persistent disease defined as WHO-CPS score ≥1) and of the proportion of patients with severe disease or who were dead at day 28 in each treatment arm (WHO-CPS score ≥6); and (3) analysis of the progression to severe respiratory failure (defined as respiratory ratio <150 mm Hg, necessitating high-flow oxygen (HFO), NIV or MV) or death by day 14. All three confirmatory analyses fully supported the clinical benefit of anakinra treatment. Compared to placebo, the unadjusted proportional odds of having a worse score on the 11-point WHO-CPS at day 14 with anakinra was 0.57 (95% CI 0.42–0.77, *P* < 0.0001) (Extended Data Fig. 1). After multivariate adjustment using the stratification factors for randomization as covariates, the OR was 0.58 (95% CI 0.42–0.79, *P* = 0.001) (Supplementary Table 1), showing that treatment with anakinra was an independent variable associated with clinical benefit compared to placebo at day 14. Multivariate logistic regression analysis of persistent disease at day 28 indicated that severe COVID-19 at baseline significantly increased the risk of disease persistence, whereas treatment with anakinra significantly reduced the risk (OR of WHO-CPS ≥1, anakinra versus placebo: 0.36; 95% CI 0.25–0.53, *P* < 0.0001) (Supplementary Table 2). In addition, treatment with anakinra significantly reduced the risk of severe disease or death at day 28 (OR of WHO-CPS ≥6, anakinra versus placebo: 0.46; 95% CI 0.26–0.83, *P =* 0.010) (Supplementary Table 2). The third confirmatory analysis validated the results of the phase 2 SAVE trial. A lower proportion of patients treated with anakinra progressed to severe respiratory failure or death by day 14 compared to placebo (20.7% versus 31.7% in the anakinra and placebo arms, respectively; hazard ratio = 0.62, 95% CI 0.45–0.87, *P* = 0.005) (Extended Data Fig. 2 and Supplementary Table 3). Finally, survival analysis showed that anakinra treatment significantly reduced the risk of death by day 28 compared to placebo (3.2% versus 6.9% in the anakinra and placebo arms, respectively; hazard ratio = 0.45, 95% CI 0.21–0.98, *P* = 0.045) (Extended Data Fig. 3).

The 11 points of the WHO-CPS are also grouped into five strata. Although, in the original protocol, it was considered to compare the frequency of strata between the two arms, the statistical analysis plan (SAP) developed with the COVID-19 Emergency Task Force (COVID-ETF) of the European Medicines Agency (EMA) pre-defined the use of the entire 11-point WHO-CPS. The comparison of outcomes using the WHO-CPS strata (uninfected (0), ambulatory with mild disease (1–3), hospitalized with mild disease (4,5), hospitalized with severe disease (6–9) and dead (10)) between the two study arms (Supplementary Tables 4 and 5) was also consistent with the analysis performed using the full 11-point scale of the WHO-CPS.

The rate of protocol deviations from the standard of care treatment was significantly greater in patients randomized to the placebo arm (27/189, 14.3% versus 13/405, 3.2% in the anakinra arm; *P* < 0.0001). Protocol deviations regarding dexamethasone use in the placebo arm were most commonly related to increasing the dose and/or lengthening the duration of administration (Supplementary Table 6). All five sensitivity analyses confirmed the analysis of the primary endpoint (Supplementary Table 7).

**Extended Data Fig. 4:**
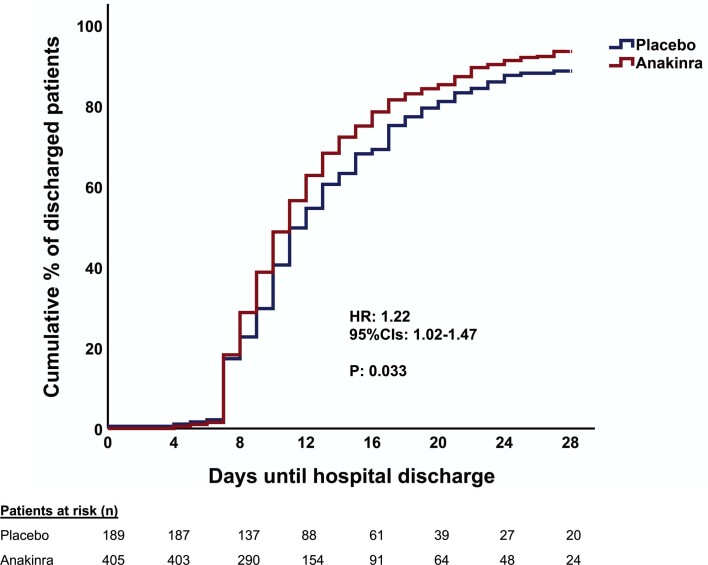
Time to hospital discharge. Comparison is done by univariate Cox regression analysis. Abbreviations: CI: confidence intervals; HR: hazard ratio; n: number. Source data

**Extended Data Fig. 5:**
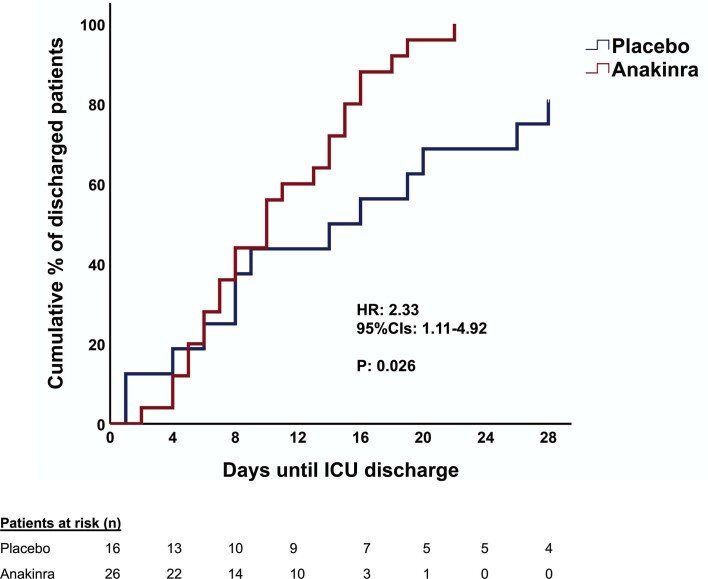
Time to discharge from the intensive care unit. Analysis involves only patients who were admitted in the intensive care unit. Comparison is done by univariate Cox regression analysis. Abbreviations: CI: confidence intervals; HR: hazard ratio; n: number. Source data

A significant benefit with anakinra treatment was observed for all five secondary clinical endpoints. Decreases in WHO-CPS score from baseline by days 28 and 14 and Sequential Organ Failure Assessment (SOFA) score from baseline by day 7 were significantly greater with anakinra versus placebo (Table 2 and Supplementary Tables 8–10). Moreover, in the anakinra group, mean time until hospital and intensive care unit (ICU) discharge was 1 and 4 d shorter, respectively (Table 2 and Extended Data Figs. 4 and 5).

**Fig. 3:**
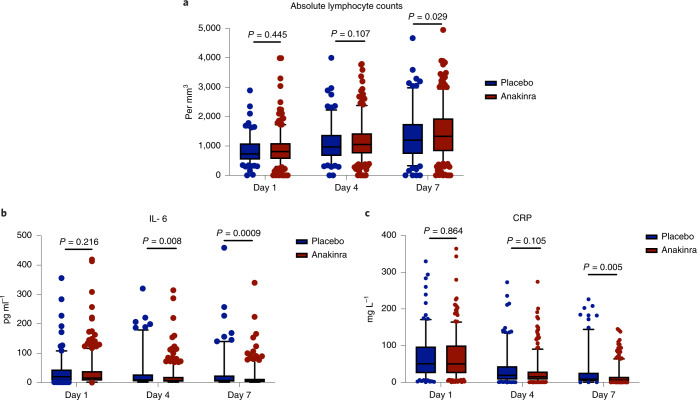
Levels of lymphocytes, IL-6 and CRP over days of follow-up. Day 1 sampling was done before start of administration of the study drug. The two-sided *P* values of comparisons for each day of follow-up are provided. The comparisons were performed by the Mann–Whitney *U* test. The lower whisker indicates the 95% lower CI; the lower bound of the box indicates the first quartile; the center of the box indicates the median; the upper bound of the box indicates the third quartile; and the upper whisker indicates the 95% upper CI. Circles represent outliers. **a**, The respective number of measurements of the absolute lymphocyte count of day 1 for placebo and anakinra were for 184 and 387 patients; minimum values were 782 and 799 per mm^3^ and maximum values were 1,008 and 1,238 per mm^3^. The respective number of measurements of the absolute lymphocyte count of day 4 for placebo and anakinra were for 176 and 386 patients; minimum values were 1,008 and 1,114 per mm^3^ and maximum values were 1,183 and 1,938 per mm^3^. The respective number of measurements of the absolute lymphocyte count of day 7 for placebo and anakinra were for 171 and 377 patients; minimum values were 1,216 and 1,386 per mm^3^ and maximum values were 1,446 and 1,551 per mm^3^. **b**, The respective number of measurements of IL-6 of day 1 for placebo and anakinra were for 182 and 394 patients; minimum values were 1.4 and 0.4 pg ml^−1^ and maximum values were 6,263.0 and 418.9 pg ml^−1^. The respective number of measurements of IL-6 of day 4 for placebo and anakinra were for 176 and 381 patients; minimum values were 1.4 and 1.4 pg ml^−1^ and maximum values were 1,352.0 and 1,209.0 pg ml^−1^. The respective number of measurements of IL-6 of day 7 for placebo and anakinra were for 167 and 376 patients; minimum values were 1.4 and 1.4 pg ml^−1^ and maximum values were 1,900.0 and 867.5 pg ml^−1^. **c**, The respective number of measurements of CRP of day 1 for placebo and anakinra were for 183 and 404 patients; minimum values were 0.9 and 0.8 mg L^−1^ and maximum values were 328.8 and 411.9 mg L^−1^. The respective number of measurements of CRP of day 4 for placebo and anakinra were for 180 and 393 patients; minimum values were 0.40 and 0.41 mg L^−1^ and maximum values were 272.3 and 274.0 mg L^−1^. The respective number of measurements of CRP of day 7 for placebo and anakinra were for 174 and 381 patients; minimum values were 0.5 and 0.1 mg L^−1^ and maximum values were 226.5 and 145.1 mg L^−1^. Source data

Over-time follow-up of laboratory values showed that, in patients who were treated with anakinra compared to patients who received placebo: (1) the absolute lymphocyte count was significantly increased by day 7; (2) circulating IL-6 levels were significantly decreased by days 4 and 7; and (3) plasma CRP levels were significantly decreased by day 7 (Fig. 3).

### Adverse events

The frequency of patients with at least one serious treatment-emergent adverse event (TEAE) was 21.7% (41/189) in the placebo arm and 16.0% (65/405) in the anakinra arm (Table 3 and Supplementary Table 11). The most common captured serious TEAEs were infections, but they were less frequent in the anakinra arm than the placebo arm (34/405, 8.4% versus 30/189, 15.9%). The next most common serious TEAEs were ventilator-associated pneumonia (9/405, 2.2% versus 15/189 7.9%); septic shock and multiple organ dysfunction (6/405, 1.5% versus 7/189, 3.7%); bloodstream infections (12/405, 3.0% versus 6/189, 3.2%); and pulmonary embolism (6/405, 1.5% versus 4/189, 2.1%). The most common non-serious TEAEs were the increase of liver function tests (145/405, 35.8% versus 63/189, 33%) and hyperglycemia (148/405, 36.5% versus 76/189, 40.2%), which were similar in frequency between the two arms. The frequency of non-serious anemia was lower in anakinra-treated patients (58/405, 14.3% versus 37/189, 19.6%). There was a trend for greater frequency of neutropenia with anakinra treatment (12/405, 3% versus 1/189, 0.5% in placebo) (Table 3 and Supplementary Table 12).

**Table 3:** Most common (>2%) TEAEs and serious TEAEs

	Placebo (*n* = 189)	Anakinra (*n* = 405)	*P* value
At least one serious TEAE, *n* (%)	41 (21.7)	65 (16.0)	0.107
Type of serious TEAEs, *n* (%)			
Infections and infestations, total	30 (15.9)	34 (8.4)	0.010
Ventilator-associated pneumonia	15 (7.9)	9 (2.2)	0.003
Septic shock and multiple organ dysfunction	7 (3.7)	6 (1.5)	0.128
Bloodstream infection	6 (3.2)	12 (3.0)	1.000
Probable hospital-acquired infection	7 (3.7)	11 (2.7)	0.608
Hospital-acquired pneumonia	5 (2.6)	6 (1.5)	0.339
Acute pyelonephritis	4 (2.1)	5 (1.2)	0.476
Pulmonary embolism	4 (2.1)	6 (1.5)	0.733
At least one non-serious TEAE, n (%)	156 (82.5)	335 (82.7)	1.00
Type of adverse event—no. (%)			
Neutropenia	1 (0.5)	12 (3.0)	0.072
Anemia	37 (19.6)	58 (14.3)	<0.0001
Thrombocytopenia	4 (2.1)	9 (2.2)	1.00
Rash at the injection site	3 (1.5)	15 (3.7)	0.203
Constipation	16 (8.5)	39 (9.6)	0.761
Diarrhea	8 (4.2)	14 (3.5)	0.645
Increase of liver function tests	63 (33.3)	145 (35.8)	0.580
Bradycardia	19 (10.1)	36 (8.9)	0.880
Headache	8 (4.2)	16 (4.0)	1.00
Anxiety	11 (5.8)	33 (8.2)	0.400
Creatinine increase	9 (4.8)	17 (4.2)	0.823
Hyperglycemia	76 (40.2)	148 (36.5)	0.413
Hyponatremia	23 (12.2)	32 (7.9)	0.175
Hypernatremia	17 (9.0)	46 (11.4)	0.474
Hypokalemia	12 (6.3)	11 (2.7)	0.040
Hyperkalemia	13 (6.9)	36 (8.9)	0.522
Hypocalcemia	20 (10.6)	32 (7.9)	0.279

Comparisons were performed with the Fisher exact test.

### Post hoc analyses

Although no differences between the two arms were found for baseline values of IL-6 (*P* = 0.22), ferritin (*P* = 0.59) and respiratory ratio (*P* = 0.10), multivariate analysis of the 28-d WHO-CPS was repeated post hoc including, as independent variables, IL-6, ferritin and respiratory ratio. For this analysis, the three variables were dichotomized using their median value. The intake of remdesivir was also included as an independent variable. Results confirmed anakinra benefit (Supplementary Table 13).

One hundred forty-six patients had low baseline CRP (below the first quartile of 25.3 mg L^−1^)^6^; several of these patients had low lymphocytes and increased IL-6, ferritin and suPAR (Supplementary Table 14). Clinical benefit of anakinra was found in patients with low CRP (Supplementary Table 15).

**Extended Data Fig. 6:**
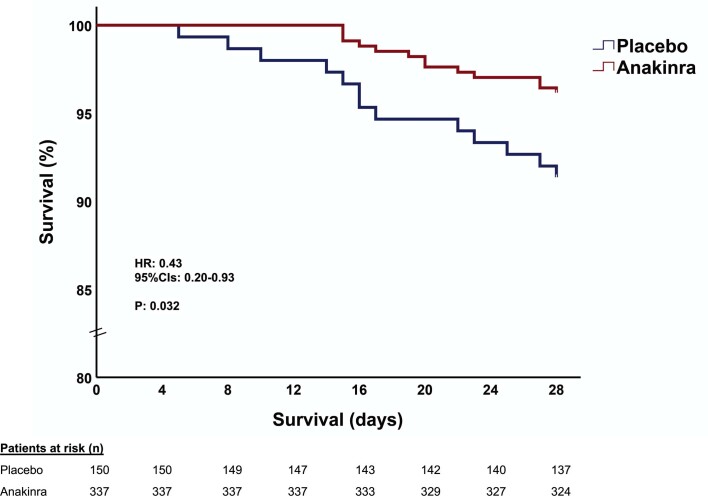
Survival analysis among patients who score 2 or more points of the COVID-associated hyperinflammation syndrome score. Comparison is done by univariate Cox regression analysis. Abbreviations: CI: confidence intervals; HR: hazard ratio; n: number. Source data

**Extended Data Fig. 7:**
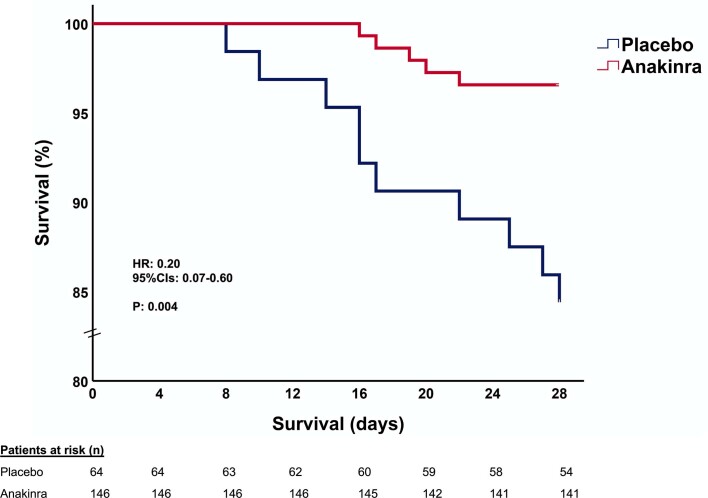
Survival analysis among patients who score positive for the criteria of prediction of cytokine storm in COVID-19. Comparison is done by univariate Cox regression analysis. Abbreviations: CI: confidence intervals; HR: hazard ratio; n: number. Source data

Four hundred eighty-seven patients had scores of 2 or more points for the COVID-associated hyper-inflammation syndrome score;^7^ multivariate ordinal regression analysis of WHO-CPS on day 28 for these patients showed overall anakinra benefit (Supplementary Table 16). A survival benefit was also found (28-d mortality of 13/337 patients, 3.9% versus 13/150 patients, 8.7%; Extended Data Fig. 6). Two hundred ten patients met the predictive criteria for cytokine storm,^8^ and an anakinra benefit was found (28-d mortality of 5/146 patients, 3.4% versus 10/64 patients, 15.6%; Supplementary Table 17 and Extended Data Fig. 7).

**Extended Data Fig. 8:**
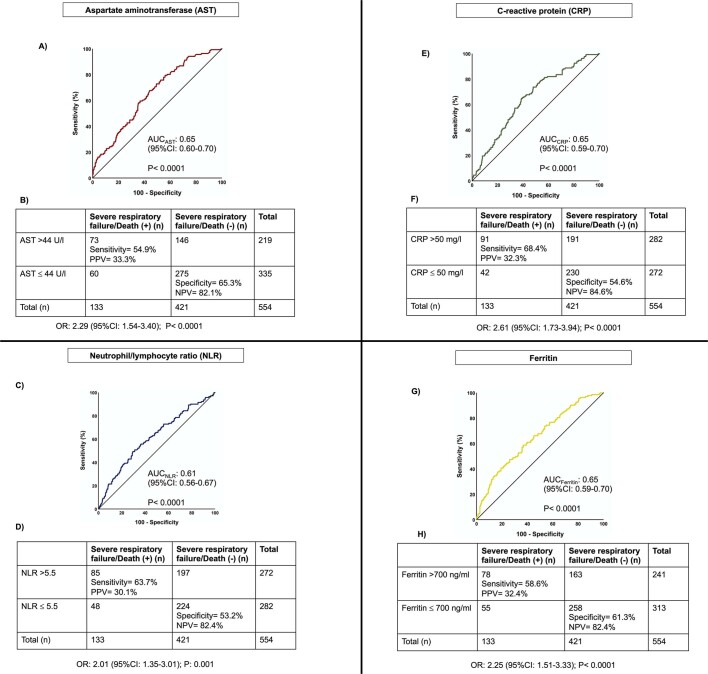
AST, NLR, CRP and ferritin for prediction of response to anakinra. A, C, E, and G) Receiver operator characteristics (ROC) curves of AST, NLR, CRP and ferritin to predict progression into severe respiratory failure (SRF) or death the first 14 days. The exact P-value of the ROC analysis in panel A is 9.3 ×10-8; in panel C is 5.1 ×10-5; in panel E is 2.7 ×10-7; and in panel G is 1.6 ×10-7. B, D, G and H) Diagnostic performance of AST > 44U/l, NLR > 5.5, CRP > 50 mg/l and ferritin > 700 ng/ml for prognostication of progression into SRF or death by day 14. The selected cut-offs are the Youden indexes for the best trade-off of sensitivity and specificity for each variable. Analysis involved patients for whom all four variables were available at baseline before start of the study drug. Comparisons are done by Mantel-Haenszel test estimating the odds ratio and 95% confidence intervals. The exact P value of the test in panel B is 4.1 ×10-5; in panel F 5.0 ×10-6; and in H 6.5 ×10-4. Abbreviations AUC: area under the curve; AST: aspartate aminotransferase; CI: confidence intervals; CRP: C-reactive protein; n: number of patients; NLR: neutrophil to lymphocyte ratio; NPV: negative predictive value; OR: odds ratio; PPV: positive predictive value. Source data

Predictors of favorable response to anakinra were defined using cutoffs of laboratory parameters that can predict progression to severe respiratory failure or death after 14 d (Extended Data Fig. 8). A combination of at least two of CRP >50 mg L^−1^, neutrophil-to-lymphocyte ratio (NLR) >5.5, ferritin >700 ng ml^−1^ and aspartate aminotransferase (AST) > 44 U L^−1^ might predict this unfavorable outcome (Supplementary Table 18). Patients with levels above these cutoffs had lower odds of progression to severe respiratory failure or death if they received anakinra (Supplementary Table 19).

We collected post hoc information for the 14-d outcome of patients with suPAR <6 ng ml^−1^; only 2.9% of patients progressed to severe respiratory failure or death.

## Discussion

The SAVE-MORE trial evaluated a novel approach for the management of COVID-19, which relies on early identification of patients at risk for unfavorable outcome using suPAR and provision of targeted treatment with anakinra. Results showed considerable efficacy of 10-d subcutaneous administration of anakinra in patients with COVID-19 with plasma suPAR ≥6 ng ml^−1^. The odds of a worse clinical outcome at day 28 with anakinra, as compared to placebo, was 0.36. The clinical benefit with anakinra treatment was already apparent from day 14, and this is of clinical importance because the first 14 d is the period during which a patient is expected to worsen; anakinra benefit was maintained until day 28. The magnitude of the efficacy of anakinra was shown in all multivariate analyses where in the presence of anakinra treatment the effect of baseline disease severity on the final outcome was lost. The proportion of patients who fully recovered exceeded 50%, and the number of patients who remained with severe disease was reduced by 54%. Most of the study population had severe COVID-19 at baseline, and 85.9% were receiving standard of care treatment containing dexamethasone. Relative decrease of mortality was 55% and reached 80% for patients likely having cytokine storm^8^. The incidence of serious TEAEs, mainly of infections, was lower in patients treated with anakinra. Patients treated with anakinra had a trend for more often non-serious neutropenia.

Two main limitations of the SAVE-MORE trial need to be acknowledged: the lack of enrollment of patients with critical COVID-19 and the difficulty for application of suPAR in all hospital settings. Not all hospitalized patients with COVID-19 have as high risk of mortality as patients progressing to critical illness, who were included in the REMAP-CAP and RECOVERY trials^9,10^. This was further confirmed in the STOP-COVID^11^ and CAN-COVID^12^ trials where the 28-d mortality of placebo-treated patients with non-critical COVID-19 ranged from 5.5% to 7.2%. The low mortality in these studies might reflect an improvement of outcomes with the current standard of care compared to the beginning of the pandemic. Results of the SAVE-MORE trial indicated that suPAR is an indicator not only of the risk of progression to severe respiratory failure or death but also of persistence of COVID-19. Most patients who received placebo remained ambulatory with symptoms on day 28. The introduction of suPAR to guide treatment could be problematic in settings where this tool is not available. Measuring suPAR is introducing a personalized treatment approach, because early increase of suPAR is indicative of excess release of DAMPs, leading to pro-inflammatory phenomena through activation of IL-1α/β^3^. Anakinra blocks both IL-1α and IL-1β by blocking their common receptor. The attenuation of the inflammatory responses by anakinra was shown by the decrease of IL-6 and of CRP circulating concentrations and by the increase of the absolute lymphocyte counts. Post hoc analysis revealed that CRP, NLR, ferritin and AST are predictors of favorable anakinra response and might also be used instead of suPAR.

CRP and ferritin are used to classify COVID-19 hyper-inflammation and work also as predictors of treatment escalation^6^. In the SAVE-MORE study, some patients enrolled because of increased suPAR had low CRP; anakinra benefit was found even when CRP was low.

The results validate the findings of the previous SAVE open-label phase 2 trial. In SAVE, the incidence of respiratory failure after 14 d with anakinra treatment was 22.3%^5^; in the SAVE-MORE trial, it was 20.7%. For patients in SAVE-MORE who were eventually admitted to the ICU, time until discharge was significantly shorter in the anakinra-treated group than in the placebo group, as was also observed in the SAVE trial^5^.

Since the beginning of the COVID-19 pandemic, immunomodulators have been suggested as a strategy to attenuate the exaggerated immune response of the host^13^. The most common administered drugs are anakinra and tocilizumab, targeting the IL-1 and IL-6 pathways, respectively. Anakinra use in COVID-19 has been reported in several retrospective and prospective studies, as well as in one randomized controlled trial^5,14–21^. Although most studies report mortality benefit, it is difficult to compare findings from observational studies with those of the SAVE-MORE trial. Previous studies differed with regard to selection of patients, severity of illness and stage of the disease. Duration of treatment, dose and route of administration were also variable, and the WHO-CPS was not studied as a primary endpoint. Indeed, four of the studies were done in patients with critical illness, with plasma levels of CRP and ferritin exceeding the levels of the SAVE-MORE study population^14–18^. In the CAN-COVID trial, 454 patients with hypoxic COVID-19 not requiring MV and with signs of hyper-inflammation were randomized to a single injection of placebo or the anti-IL-1β monoclonal antibody canakinumab^12^. The trial failed to meet the primary endpoint—namely, the rate of survivors not in need of NIV or MV by day 29. The differential findings of SAVE-MORE could be explained by the activity of anakinra against IL-1α, which is not inhibited by canakinumab. An alternative explanation for this discrepancy is that patient stratification in CAN-COVID used CRP or ferritin instead of suPAR as biomarkers of hyper-inflammation.

The clinical benefit of tocilizumab has been studied in six clinical trials. Τhe patient populations of four clinical trials were much similar to the population of the SAVE-MORE trial^22–25^. Clinical benefit from tocilizumab treatment was shown in only one trial^23^. Conversely, clinical benefit from tocilizumab treatment was found in the RECOVERY^9^ and REMAP-CAP^10^ trials, which included patients with severe and critical COVID-19. In the RECOVERY trial^9^, mortality was decreased from 35% with usual care to 31%, and, in the REMAP-CAP trial^10^, the median number of organ support-free days was increased from 0 d with usual care to 10 d with tocilizumab treatment. The benefit of tocilizumab in patients with more severe disease might be explained by the biology of the disease course. We previously showed that circulating monocytes in critical COVID-19 present with complex immune dysregulation characterized by decreased efficiency of antigen presentation and inappropriate maintenance of the potential for excess cytokine production, which were restored upon exposure to tocilizumab^26^. Our findings suggest that suPAR should be measured upon admission of all patients with COVID-19 who do not need oxygen or who need nasal or mask oxygen, and that, if suPAR levels are 6 ng ml^−1^ or higher, anakinra treatment might be a suitable therapy. For patients with low respiratory ratio who need NIV or MV, tocilizumab might be the most appropriate drug of choice.

In conclusion, the SAVE-MORE trial showed that early start of treatment with anakinra guided by suPAR levels in patients hospitalized with moderate and severe COVID-19 significantly reduced the risk of worse clinical outcome at day 28.

## Methods

### Trial oversight

SAVE-MORE is a prospective, double-blind randomized clinical trial conducted in 37 study sites (29 in Greece and eight in Italy). The protocol (available with the full text of this article) was finalized after advice from the COVID-ETF of the EMA (document EMA/659928/2020). The full SAP was developed through interactions with the EMA COVID-ETF before database lock; no amendment was needed.

The protocol was approved by the National Ethics Committee of Greece (approval 161/20) and by the Ethics Committee of the National Institute for Infectious Diseases Lazzaro Spallanzani, IRCCS, in Rome (1 February 2021) (EudraCT no. 2020-005828-11; ClinicalTrials.gov NCT04680949). The trial was sponsored by the Hellenic Institute for the Study of Sepsis (HISS) and funded, in part, by HISS and, in part, by Swedish Orphan Biovitrum (Sobi). HISS was responsible for the design, conduct, analysis and interpretation of data and the decision to publish. The laboratory of Immunology of Infectious Diseases of the 4th Department of Internal Medicine at ATTIKON University General Hospital served as the central laboratory. The data lock for all events until day 28 was done on 29 April 2021.

### Patients

Inclusion criteria were as follows: (1) adult patients of either sex; (2) for women, unwillingness to remain pregnant during the study period; (3) confirmed infection by SARS-CoV-2 by molecular test; (4) findings in chest X-ray or chest computed tomography compatible with lower respiratory tract infection; (5) need for hospitalization; and (6) plasma suPAR ≥6 ng ml^−1^. Exclusion criteria were as follows: (1) any stage IV malignancy; (2) any do-not-resuscitate order; (3) ratio or partial oxygen pressure to fraction of inspired oxygen less than 150 mmHg; (4) need of NIV (CPAP or BPAP) or MV; (5) any primary immunodeficiency; (6) fewer than 1,500 neutrophils per mm^3^; (7) oral or intravenous intake of corticosteroids at a daily dose greater than or equal to 0.4 mg kg^−1^ of prednisone for a period longer than the last 15 d; (8) any anti-cytokine biological treatment, including JAK inhibitors, during the last 1 month; (9) severe hepatic failure; (10) end-stage renal failure necessitating hemofiltration or peritoneal hemodialysis; and (11) pregnancy or lactation. All patients or their legal representatives provided written informed consent before enrollment.

### Trial interventions

Patients meeting all inclusion criteria and not meeting any exclusion criterion were subject to blood draw. suPAR was measured in plasma using the suPARnostic Quick Triage kit (Virogates) and a point-of-care reader. Patients with suPAR 6 ng ml^−1^ or higher were electronically 1:2 randomized into treatment with placebo or anakinra using four randomization strata: classification into moderate or severe disease using the WHO definition based on the need for oxygen support (https://www.who.int/publications/i/item/clinical-management-of-covid-19); need for dexamethasone intake; body mass index (BMI) higher than 30 kg m^−^^2^; and country. The 1:2 randomization was agreed with by the COVID-ETF of the EMA, taking into consideration the beneficial effect shown for anakinra in the phase 2 SAVE trial^5^, in the attempt to expose as few patients as possible to placebo. The study drug was administered subcutaneously once daily in the thigh or the abdomen for 7–10 d. The dose regimen and duration of treatment were based on the beneficial results of the same regimen in the phase 2 SAVE trial^5^. Patients allocated to placebo treatment were daily injected with 0.67 ml of 0.9% sodium chloride; patients allocated to the active drug were daily injected with 100 mg of anakinra at a final volume of 0.67 ml. The study drug was prepared by an unblinded pharmacist with access to the electronic study system using a separate username and password. Administration was done by a blinded study nurse. All patients were receiving pre-defined standard of care, which consisted of regular monitoring of physical signs, oximetry and anti-coagulation. Patients with severe disease by the WHO definition (https://www.covid19treatmentguidelines.nih.gov/concomitant-medications) were also receiving 6 mg of dexamethasone intravenously daily for 10 d. Remdesivir treatment was left at the discretion of the attending physicians; other biologicals targeting cytokines and kinase inhibitors were not allowed. All patients were allowed support with NIV or MV if needed.

Study visits were done daily for 10 d, on day 14 and on day 28. At each study visit, the following were recorded: non-serious and serious TEAEs, WHO-CPS score, SOFA score and co-administered treatment. Visits were done by phone for patients discharged by day 7. Data were captured after review of all medical and nursing charts by a physician team blinded to the allocation group. Blood samples and nasopharyngeal swabs were collected before start of the study drug and at days 4 and 7 for the measurement of biomarkers.

All serious and non-serious TEAEs were graded according to the Common Terminology Criteria for Adverse Events (v5.0).

### Endpoints

The primary study endpoint was the overall comparison of the distribution of frequencies of the scores from the 11-point WHO-CPS between the two arms of treatment on day 28. Secondary endpoints included the changes of WHO-CPS scores at days 14 and 28 from the baseline (before start of the study drug); the change of SOFA score at day 7 from baseline; the time until hospital discharge; the time of stay in the ICU for patients eventually admitted to the ICU; and the comparison of biomarkers. The outcome by day 14 of patients excluded from enrollment because of suPAR lower than 6 ng ml^−1^ was captured post hoc.

### Statistical analysis

The primary effect size was calculated based on the finding from the phase 2 SAVE trial^5^, in which there was a 25% difference between the two treatment arms in the number of patients who had scores of 6 or higher on the WHO-CPS by day 28 (42% of comparators versus 16.3% of anakinra-treated patients). To replicate this primary effect size in the SAVE-MORE trial, and with a 90% power at the 5% significance level, a sample size of 200 was needed for the placebo treatment arm and 400 for the anakinra treatment arm. Data were analyzed for the ITT population. Missing data were imputed by last observation carried forward (LOCF). WHO-CPS is an ordinal 11-point variable ranging from 0 to 10, and comparisons were done by univariate and multivariate ordinal regression analysis using the logit function. Results were expressed as the ORs and 95% CIs. The two basic assumptions of the model—namely, proportional odds and the goodness-of-fit test—were checked. According to advice from the EMA COVID-ETF, the variables used for stratified randomization—that is, disease severity, intake of dexamethasone, BMI higher than 30 kg m^−^^2^ and country—were entered as covariates in the multivariate model. According to the same advice, the analysis of the primary endpoint should have been supported by three analyses: comparison of WHO-CPS scores by day 14; logistic regression analysis separately for patients at the two spectra of the WHO-CPS at day 28; and time of progression to respiratory failure by day 14. The first spectrum of the WHO-CPS was defined as patients fully recovered with negative viral load (WHO-CPS 0 points); patients with persistent disease scored from 1 to 10 points on the WHO-CPS. The second spectrum was defined as patients with severe disease or who were dead scoring 6 or more points on the WHO-CPS, contrary to non-severe patients scoring 5 or fewer points. Time of progression to severe respiratory failure or death was compared by forward stepwise Cox regression analysis. Logistic stepwise analysis was used for the two spectra of the WHO-CPS. Survival analysis was done using Cox regression analysis. Five sensitivity analyses were conducted to assess robustness: exclusion of population deviating from the standard of care; population receiving at least seven doses of the study drug; complete analysis set; responder analysis treating missing values as non-responders; and comparison of the unadjusted and adjusted treatment effects. Analysis was conducted using IBM SPSS Statistics v26.0. All *P* values were two sided, and any *P* value less than 0.05 was considered statistically significant. The complete SAP is provided in the Supplementary Appendix. Although, in the original protocol, it was considered to compare the frequency of strata between the two arms, the SAP developed with the COVID-ETF pre-defined the use of the entire 11-point WHO-CPS before the database lock.

### Reporting Summary

Further information on research design is available in the Nature Research Reporting Summary linked to this article.

## Online content

Any methods, additional references, Nature Research reporting summaries, source data, extended data, supplementary information, acknowledgements, peer review information; details of author contributions and competing interests; and statements of data and code availability are available at 10.1038/s41591-021-01499-z.

## Supplementary information

Supplementary Information Supplementary Tables 1–19

Reporting Summary

## Data Availability

Requests for de-identified patient data by researchers with proposed use of the data can be made to the corresponding author with specific data needs, analysis plans and dissemination plans. Those requests will be reviewed by a study steering committee and the study sponsor for release upon publication. Contact: egiamarel@med.uoa.gr. Source data are provided with this paper.

## Source data

Source Data Fig. 1
Source Data Fig. 2
Source Data Fig. 3
Source Data Extended Data Fig. 1
Source Data Extended Data Fig. 2
Source Data Extended Data Fig. 3
Source Data Extended Data Fig. 4
Source Data Extended Data Fig. 5
Source Data Extended Data Fig. 6
Source Data Extended Data Fig. 7
Source Data Extended Data Fig. 8
