# Supplementary material for: Early treatment of COVID-19 with anakinra guided by soluble urokinase plasminogen receptor plasma levels: a double-blind, randomized controlled phase 3 trial
Source: Nat Med. 2021 Sep 3;27(10):1752–60. doi: 10.1038/s41591-021-01499-z (PMC8516650; doi:10.1038/s41591-021-01499-z)
Supplement: Supplementary file 1 — Supplementary Tables 1–19 [file 41591_2021_1499_MOESM1_ESM.pdf]

nature portfolio

https://doi.org/10.1038/s41591-021-01499-z

Supplementary information

Early treatment of COVID-19 with anakinra guided by soluble urokinase plasminogen receptor plasma levels: a double-blind, randomized controlled phase 3 trial

In the format provided by the authors and unedited

Supplementary information

Early treatment of COVID-19 with anakinra guided by soluble urokinase plasminogen receptor plasma levels: a double-blind, randomised controlled phase 3 trial

Table of contents

Content	Page
Supplementary Table 1. First confirmatory analysis of the primary endpoint	2
Supplementary Table 2. Second confirmatory analyses of the primary endpoint	3
Supplementary Table 3. Third confirmatory analysis of the primary endpoint	4
Supplementary Table 4. Distribution of strata of the primary study endpoint	5
Supplementary Table 5 Analysis of strata of the primary study endpoint	6
Supplementary Table 6. Recorded deviations from the per-protocol standard-of-care treatment	7
Supplementary Table 7. The five sensitivity analyses for the primary study endpoint	8
Supplementary Table 8. Changes of the World Health Organization Clinical Progression Scale (WHO-CPS) at day 28 from baseline	9
Supplementary Table 9. Changes of the World Health Organization Clinical Progression Scale (WHO-CPS) by day 14 from baseline	11
Supplementary Table 10. Changes of the Sequential Organ Failure Assessment score at day 7 from baseline	12
Supplementary Table 11. Complete list of serious treatment-emergent adverse events (TEAE) Classified by System	13
Supplementary Table 12. Complete list of non-serious treatment-emergent adverse events (TEAE) Classified by System	15
Supplementary Table 13 Univariate and multivariate ordinal regression analysis of the WHO-CPS on day 28.	17
Supplementary Table 14 Baseline absolute lymphocyte counts and concentrations of ferritin, IL-6 and suPAR among patients with low baseline CRP	18
Supplementary Table 15 Univariate and multivariate ordinal regression analysis of the WHO-CPS on day 28 among patients with low CRP.	19
Supplementary Table 16 Univariate and multivariate ordinal regression analysis of the WHO-CPS on day 28 among patients with COVID-associated hyperinflammatory syndrome (cHIS).	20
Supplementary Table 17 Univariate and multivariate ordinal regression analysis of the WHO-CPS on day 28 among patients positive according to the predictive criteria for progression into with COVID-associated cytokine storm	21
Supplementary Table 18 Incidence of severe respiratory failure (SRF) and/or death until day 14 for patients scoring for at least two of AST, CRP, ferritin and NLR above the defined cut-offs	22
Supplementary Table 19 Incidence of severe respiratory failure (SRF) and/or death until day 28 according to risk as this is defined by AST, CRP, ferritin and NLR before start of the study drug separately for patients allocated to the SoC and placebo group and for patients allocated to the SoC and Anakinra group	23

Supplementary Table 1 First confirmatory analysis of the primary endpoint. The comparisons between anakinra and placebo for the primary study endpoint (World Health Organization Clinical Progression Scale) on day 14 are done by univariate and multivariate ordinal regression analyses. Co-variates entered in the multivariate analysis were those used for stratified randomization according to the received advice by the COVID-ETF of the EMA.

**Variable**	**Univariate analysis**			**Multivariate analysis**		
	Odds ratio	95% CIs	P-value	Odds ratio	95% CIs	P-value
Group of treatment (Anakinra vs placebo)	0.57	0.42-0.77	<0.0001	0.58	0.42-0.79	0.001
Intake of dexamethasone (Yes/No)	2.23	1.53-3.26	<0.0001	1.69	0.70-4.11	0.242
Severe COVID-19 by WHO (Yes/No)	2.23	1.53-3.26	<0.0001	1.36	0.56-3.27	0.493
BMI >30 kg/m^2 (Yes/No)	1.15	0.86-1.56	0.343	1.05	0.78-1.42	0.717
Country (Italy vs Greece)	1.14	0.72-1.79	0.572	1.26	0.79-2.00	0.320

BMI: body mass index; CI: confidence internal; WHO: World Health Organization

Supplementary Table 2 Second confirmatory analyses of the primary endpoint. The comparisons between anakinra and placebo for the two spectra of the scale of the WHO Clinical Progression Scale-WHO-CPS) on day 28 are done by univariate and multivariate step-wise logistic regression analyses. The first spectrum involves patients with fully resolved disease (scoring 0 points of the WHO-CPS) or persistent disease (scoring 1 to 10 points of the WHO-CPS). The second spectrum involves patients with severe disease or dead (scoring 6 to 10 points of the WHO-CPS) or without severe disease (scoring 0 to 5 points of the WHO-CPS). Co-variates entered in the multivariate model were those used for stratified randomization according to the received advice by the COVID-ETF of the EMA. The exact P-value of the anakinra vs placebo comparison in the multivariate analysis for towards fully resolved or persistent disease is 1.4 x 10^{-7}.

**Variable**			**Univariate analysis**		**Multivariate analysis**	
	**Analysis towards fully resolved (WHO-CPS =0 points) or persistent disease (WHO-CPS ≥1 point)**					
	**Fully resolved (n= 254)**	**Persistence (n= 340)**	**Odds ratio (95% CIs)**	**P-value**	**Odds ratio (95% CIs)**	**P-value**
Anakinra treatment, n (%)	204 (80.3)	201 (59.1)	0.35 (0.23-0.52)	<0.0001	0.36 (0.25-0.53)	<0.0001
Intake of dexamethasone, n (%)	198 (78.0)	288 (84.7)	1.56 (1.03-2.38)	0.036	**	
Severe COVID-19 by WHO, n (%)	196 (77.2)	289 (85.0)	1.68 (1.10-2.55)	0.015	1.58 (1.02-2.42)	0.037
BMI >30 kg/m^2, n (%)	87 (34.3)	129 (37.9)	1.17 (0.84-1.65)	0.355	**	
Patients in Italy, n (%)	30 (11.8)	36 (10.6)	0.91 (0.55-1.52)	0.723	**	
	**Analysis towards severe disease/death (WHO-CPS ≥6) or no severe disease (WHO-CPS ≤5)**					
	**WHO-CPS ≤5 (n= 543)**	**WHO-CPS ≥6 (n= 51)**	**Odds ratio (95% CIs)**	**P-value**	**Odds ratio (95% CIs)**	**P-value**
Anakinra treatment, n (%)	379 (69.8)	26 (50.1)	0.45 (0.25-0.80)	0.007	0.46 (0.26-0.83)	0.010
Intake of dexamethasone, n (%)	435 (80.1)	51 (100)	*	<0.0001	**	
Severe COVID-19 by WHO, n (%)	434 (79.9)	51 (100)	*	<0.0001	**	
BMI >30 kg/m^2 n (%)	199 (36.6)	17 (33.1)	0.81 (0.44-1.50)	0.809	**	
Patients in Italy, n (%)	59 (10.9)	7 (13.7)	1.57 (0.70-3.49)	0.274	**	

*cannot be computed because one value is zero; **variables not included in equation after 2 steps of forward analysis; BMI: body mass index; CI: confidence internal; WHO: World Health Organization

Supplementary Table 3 Third confirmatory analysis of the primary endpoint
The comparisons between anakinra and placebo for progression into respiratory failure and/or death the first 14 days are done by univariate and multivariate step-wise Cox regression analyses. These analyses are done to validate the results of the phase 2 study SAVE. Respiratory failure is defined as any respiratory ratio less than 150 requiring the use of high-flow oxygen/non-invasive ventilation/mechanical ventilation or death. Co-variates entered in the multivariate model were those used for stratified randomization according to the received advice by the COVID-ETF of the EMA.

Variable	Respiratory failure		Univariate analysis		Multivariate analysis	
	**No (n= 450)**	**Yes (n= 144)**	**Hazard ratio (95% CIs)**	**P-value**	**Hazard ratio (95% CIs)**	**P-value**
Anakinra treatment, n (%)	321 (71.3)	84 (58.3)	0.62 (0.45-0.87)	0.005	0.67 (0.47-0.93)	0.017
Intake of dexamethasone, n (%)	345 (76.7)	141 (97.9)	11.63 (3.70-36.49)	<0.0001	*	
Severe COVID-19 by WHO n (%)	343 (76.2)	152 (98.6)	18.27 (4.52-73.77)	<0.0001	17.16 (4.25-69.33)	<0.0001
BMI >30 kg/m^2, n (%)	158 (35.1)	58 (40.3)	0.84 (0.60-1.17)	0.306	*	
Patients in Italy, n (%)	39 (8.7)	28 (19.4)	2.34 (1.55-3.54)	<0.001	2.21 (1.46-3.34)	<0.0001

*variables not included in equation after 3 steps of forward analysis
BMI: body mass index; CI: confidence internal; WHO: World Health Organization

Supplementary Table 4 Distribution of strata of the primary study endpoint
For this analysis, the WHO-CPS is divided into the five following strata: fully recovered PCR(-) scoring 0 points of the WHO-CPS; ambulatory with symptoms scoring 1 to 3 points of the WHO-CPS; hospitalized with moderate disease scoring 4 to 5 points of the WHO-CPS; hospitalized with severe disease scoring 6 to 9 points of the WHO-CPS; and dead scoring 10 points of the WHO-CPS

	**Placebo (N=189)**	**Anakinra (N=405)**
Fully recovered PCR(-), n (%)	50 (26.5)	204 (50.4)
Ambulatory with symptoms, n (%)	101 (53.4)	158 (39.0)
Hospitalized with moderate disease, n (%)	13 (6.9)	17 (4.2)
Hospitalized with severe disease, n(%)	12 (6.3)	13 (3.2)
Dead, n (%)	13 (6.9)	13 (3.2)

Supplementary Table 5 Analysis of strata of the primary study endpoint
The comparisons between anakinra and placebo for the primary study endpoint (World Health Organization Clinical Progression Scale, WHO-CPS) on day 28 are done by univariate and multivariate ordinal regression analyses. Co-variates entered in the multivariate model were those used for stratified randomization according to the received advice by the COVID-ETF of the EMA. For this analysis, the WHO-CPS is divided into the five following strata: fully recovered PCR(-) scoring 0 points of the WHO-CPS; ambulatory with symptoms scoring 1 to 3 points of the WHO-CPS; hospitalized with moderate disease scoring 4 to 5 points of the WHO-CPS; hospitalized with severe disease scoring 6 to 9 points of the WHO-CPS; and dead scoring 10 points of the WHO-CPS. The exact P-value of the comparison of anakinra vs placebo of the multivariate analysis is 5.6 $x10^{-8}$.

**Variable**	**Univariate analysis**			**Multivariate analysis**		
	Odds ratio	95% CIs	P-value	Odds ratio	95% CIs	P-value
Group of treatment (Anakinra vs placebo)	0.39	0.28-0.54	<0.0001	0.39	0.28-0.55	<0.0001
Intake of dexamethasone (Yes/No)	1.90	1.27-2.86	0.002	1.32	0.51-3.46	0.57
Severe COVID-19 by WHO (Yes/No)	1.98	1.32-2.97	0.001	1.47	0.57-3.83	0.42
BMI >30 kg/m^2 (Yes/No)	1.16	0.84-1.59	0.37	1.09	0.79-1.50	0.61
Country (Italy vs Greece)	1.06	0.65-1.72	0.82	1.11	0.67-1.84	0.67

BMI: body mass index; CI: confidence internal; WHO: World Health Organization

Supplementary Table 6 Recorded deviations from the per-protocol standard-of-care treatment

Deviation, n (%)	Placebo (n=189)	Anakinra (n= 405)	P-value
Administration of dexamethasone for more than 10 days	9 (4.8)	0 (0)	<0.0001
Daily dosing of dexamethasone 10-20mg	3 (1.6)	2 (0.5)	0.33
Administration of dexamethasone 6-18 mg daily with MTP	4 (2.1)	0 (0)	0.010
Stop of study drug, administration of TCZ + IVIG+ ANA	3 (1.6)	0 (0)	0.031
Administration of secukinumab	1 (0.5)	0 (0)	0.32
Dexamethasone administration in moderate disease	2 (1.1)	3 (0.7)	0.66
Dexamethasone administration for 11 days and co-administration of TCZ and MTP	0 (0)	1 (0.2)	1.00
Administration of dexamethasone for less than 10 days	1 (0.5)	1 (0.2)	0.54
Premature stop of study drug due to leukopenia, n (%)	1 (0.5)	1 (0.2)	0.54
Premature stop of study drug due to increase of aminotransferases	1 (0.5)	2 (0.5)	1.00
Premature stop of study drug due to earlier hospital discharge	1 (0.5)	2 (0.5)	1.00
Premature stop of study drug by the attending physicians after ICU admission	1 (0.5)	1 (0.2)	0.54

ANA: anakinra; ICU: intensive care unit; IVIG: intravenous γ-globulin; MTP: methylprednisolone; TCZ: tocilizumab

Supplementary Table 7 The five sensitivity analyses for the primary study endpoint
In the first four sensitivity analyses comparisons between anakinra and placebo of the primary study endpoint (World Health Organization Clinical Progression Scale) on day 28 are done by univariate and multivariate ordinal regression analyses. Co-variates entered in the multivariate model were those used for stratified randomization according to the received advice by the COVID-ETF of the EMA. The exact P-values of the comparison of anakinra vs placebo of the multivariate analyses are: a) 1.8 x10^{-9} for sensitivity analysis 1; b) 1.2 x 10^{-8} for sensitivity analysis 2; c) 4.3 x 10^{-10} for sensitivity analysis 3; and d) 2.4 x 10^{-10}.

	**Univariate analysis**			**Multivariate analysis**		
	**Sensitivity analysis 1: Per-protocol (Placebo= 162; Anakinra= 292)**					
	**Odds ratio**	**95% CIs**	**P-value**	**Odds ratio**	**95% CIs**	**P-value**
Group of treatment (Anakinra vs placebo)	0.34	0.24-0.48	<0.0001	0.35	0.25-0.48	<0.0001
Intake of dexamethasone (Yes/No)	1.72	1.15-2.59	0.008	1.42	0.47-4.20	0.530
Severe COVID-19 by WHO (Yes/No)	1.76	1.17-2.67	0.006	1.22	0.41-3.68	0.705
BMI >30 kg/m^2 (Yes/No)	1.16	0.84-1.59	0.311	1.111	0.80-1.53	0.473
Country (Italy vs Greece)	1.05	0.65-1.71	0.800	1.15	0.70-1.88	0.572
	**Sensitivity analysis 2: Population receiving ≥7 doses of the study drug (Placebo= 177; Anakinra= 382)**					
	**Odds ratio**	**95% CIs**	**P-value**	**Odds ratio**	**95% CIs**	**P-value**
Group of treatment (Anakinra vs placebo)	0.37	0.28-0.52	<0.0001	0.38	0.27-0.53	<0.0001
Intake of dexamethasone (Yes/No)	1.90	1.27-2.86	0.002	1.14	0.42-3.11	0.795
Severe COVID-19 by WHO (Yes/No)	2.03	1.36-3.05	0.001	1.70	0.63-4.58	0.292
BMI >30 kg/m^2 (Yes/No)	1.19	0.88-1.64	0.258	1.11	0.80-1.53	0.512
Country (Italy vs Greece)	1.21	0.74-1.99	0.443	1.26	0.75-2.10	0.375

BMI: body mass index; CI: confidence internal; WHO: World Health Organization.

Supplementary Table 7. The five sensitivity analyses for the primary study endpoint (continued)

	**Univariate analysis**			**Multivariate analysis**		
	**Sensitivity analysis 3: Complete cases analysis (Placebo= 188; Anakinra= 405)**					
	**Odds ratio**	**95% CIs**	**P-value**	**Odds ratio**	**95% CIs**	**P-value**
Group of treatment (Anakinra vs placebo)	0.35	0.26-0.49	<0.0001	0.36	0.26-0.49	<0.0001
Intake of dexamethasone (Yes/No)	1.91	1.28-2.84	0.001	1.51	0.59-2.83	0.278
Severe COVID-19 by WHO (Yes/No)	1.96	1.32-2.92	0.001	1.29	0.51-3.27	0.771
BMI >30 kg/m^2 (Yes/No)	1.19	0.89-1.63	0.243	1.12	0.82-1.52	0.490
Country (Italy vs Greece)	1.21	0.75-1.95	0.426	1.27	0.78-2.08	0.315
	**Sensitivity analysis 4: Responder analysis treating missing values as failures (Placebo= 189; Anakinra= 405)**					
	**Odds ratio**	**95% CIs**	**P-value**	**Odds ratio**	**95% CIs**	**P-value**
Group of treatment (Anakinra vs placebo)	0.35	0.25-0.48	<0.0001	0.36	0.26-0.49	<0.0001
Intake of dexamethasone (Yes/No)	1.92	1.29-2.85	0.001	1.49	0.59-3.79	0.278
Severe COVID-19 by WHO (Yes/No)	1.97	1.32-2.93	0.001	1.30	0.52-3.28	0.758
BMI >30 kg/m^2 (Yes/No)	1.21	0.89-1.65	0.208	1.14	0.83-1.55	0.420
Country (Italy vs Greece)	1.15	0.72-1.84	0.551	1.21	0.74-1.96	0.422
	**Sensitivity analysis 5: Comparison of the unadjusted and the adjusted model**					
	**Unadjusted**			**Adjusted**		
	**Odds ratio**	**95% CI**		**Odds ratio**	**95% CI**	**P-value**
Group of treatment (Anakinra vs placebo)	0.36	0.26-0.49		0.36	0.25-0.50	1.00

BMI: body mass index; CI: confidence internal; WHO: World Health Organization.

Supplementary Table 8 Changes of the World Health Organization Clinical Progression Scale (WHO-CPS) at day 28 from baseline
Comparisons between anakinra and placebo are done by univariate and multivariate ordinal regression analyses. Co-variates entered in the multivariate model were those used for stratified randomization according to the received advice by the COVID-ETF of the EMA. The exact P-value of the comparison of anakinra vs placebo of the multivariate analyses is 1.4×10^{-8}.

Variable	Univariate analysis			Multivariate analysis		
	Odds ratio	95% CIs	P-value	Odds ratio	95% CIs	P-value
Group of treatment (Anakinra vs placebo)	0.40	0.29-0.55	<0.0001	0.40	0.29-0.55	<0.0001
Intake of dexamethasone (Yes/No)	1.00	0.69-1.46	0.964	0.94	0.38-2.31	0.897
Severe COVID-19 by WHO (Yes/No)	1.07	0.73-1.55	0.737	1.07	0.43-2.60	0.892
BMI >30 kg/m^2 (Yes/No)	1.08	0.79-1.46	0.620	1.02	0.75-1.39	0.882
Country (Italy vs Greece)	1.64	1.03-2.64	0.036	1.65	1.02-2.67	0.040

BMI: body mass index; CI: confidence internal; WHO: World Health Organization

Supplementary Table 9 Changes of the World Health Organization Clinical Progression Scale (WHO-CPS) by day 14 from baseline
Comparisons between anakinra and placebo are done by univariate and multivariate ordinal regression analyses are done by univariate and multivariate ordinal regression analyses. Co-variates entered in the multivariate model were those used for stratified randomization according to the received advice by the COVID-ETF of the EMA.

**Variable**	**Univariate analysis**			**Multivariate analysis**		
	**Odds ratio**	**95% CIs**	**P-value**	**Odds ratio**	**95% CIs**	**P-value**
Group of treatment (Anakinra vs placebo)	0.63	0.46-0.85	0.003	0.63	0.46-0.86	0.003
Intake of dexamethasone (Yes/No)	1.28	0.88-1.85	0.199	1.19	0.49-2.88	0.689
Severe COVID-19 by WHO (Yes/No)	1.30	0.90-1.89	0.161	1.10	0.46-2.64	0.823
BMI >30 kg/m^2 (Yes/No)	1.07	0.80-1.45	0.621	1.01	0.75-1.37	0.917
Country (Italy vs Greece)	1.50	0.95-2.37	0.078	1.56	0.98-2.49	0.058

BMI: body mass index; CI: confidence internal; WHO: World Health Organization

Supplementary Table 10 Changes of the Sequential Organ Failure Assessment score at day 7 from baseline
Comparisons between anakinra and placebo are done by univariate and multivariate ordinal regression analyses. Co-variates entered in the multivariate model were those used for stratified randomization according to the received advice by the COVID-ETF of the EMA. The analysis involves patients who remained hospitalized by day 7.

**Variable**	**Univariate analysis**			**Multivariate analysis**		
	**Odds ratio**	**95% CIs**	**P-value**	**Odds ratio**	**95% CIs**	**P-value**
Group of treatment (Anakinra vs placebo)	0.63	0.46-0.86	0.004	0.64	0.47-0.88	0.007
Intake of dexamethasone (Yes/No)	1.11	0.76-1.62	0.582	0.58	0.24-1.46	0.254
Severe COVID-19 by WHO (Yes/No)	1.25	0.86-1.81	0.250	1.89	0.76-4.65	0.167
BMI >30 kg/m^2 (Yes/No)	1.25	0.92-1.69	0.146	1.26	0.92-1.71	0.143
Country (Italy vs Greece)	0.94	0.58-1.52	0.806	0.88	0.55-2.34	0.638

BMI: body mass index; CI: confidence internal; WHO: World Health Organization

Supplementary Table 11. Complete list of serious treatment-emergent adverse events (TEAE) Classified by System

	Placebo (n=189)	Anakinra (n=405)	P-value
At least one serious TEAE, n (%)	41 (21.7)	65 (16.0)	0.107
Type of serious TEAE, n (%)			
Infections and infestations, total	30 (15.9)	34 (8.5)	0.010
Ventilator-associated pneumonia	15 (7.9)	9 (2.2)	0.003
Related to the study drug	2 (1.1)	0 (0)	
Bloodstream infection	6 (3.2)	12 (3.0)	1.00
Related to the study drug	0 (0)	0 (0)	
Clostridioides difficile infection	2 (1.0)	0 (0)	0.10
Related to the study drug	0 (0)	0 (0)	
Septic Shock and multiple organ dysfunction	7 (3.7)	6 (1.5)	0.128
Related to the study drug	1 (0.5)	1 (0.2)	
Probable hospital-acquired infections	7 (3.7)	11 (2.7)	0.608
Related to the study drug	1 (0.5)	1 (0.2)	
Hospital-acquired pneumonia	5 (2.6)	6 (1.5)	0.339
Related to the study drug	1 (0.5)	0 (0)	
Acute pyelonephritis	4 (2.1)	5 (1.2)	0.476
Related to the study drug	1 (0.5)	1 (0.2)	
Intrabdominal infection	1 (0.5)	2 (0.5)	1.00
Related to the study drug	0 (0)	0 (0)	
Diagnosis of chronic hepatitis B	0 (0)	1 (0.2)	1.00
Related to the study drug	0 (0)	0 (0)	
Lung empyema	1 (0.5)	0 (0)	0.318
Related to the study drug	0 (0)	0 (0)	
New hospital admissions	1 (0.5)	0 (0)	0.318
Related to the study drug	0 (0)	0 (0)	
Acute kidney injury	1 (0.5)	3 (0.7)	1.00
Related to the study drug	0 (0)	1 (0.2)	
Anaphylactic shock	0 (0)	1 (0.2)	1.00
Related to the study drug	0 (0)	0 (0)	
Lung, heart and vessels			
Pulmonary embolism	4 (2.1)	6 (1.5)	0.733
Related to the study drug	0 (0)	0 (0)	
Vascular thrombosis	0 (0)	1 (0.2)	1.00
Related to the study drug	0 (0)	0 (0)	
Pneumomediastinum	2 (1.1)	3 (0.7)	0.655
Related to the study drug	0 (0)	0 (0)	
Pneumothorax	2 (1.1)	1 (0.2)	0.239
Related to the study drug	0 (0)	0 (0)	
Pulmonary fibrosis	0 (0)	1 (0.2)	1.00
Related to the study drug	0 (0)	0 (0)	
Lung hemorrhage	1 (0.5)	0 (0)	0.318
Related to the study drug	0 (0)	0 (0)	
Sinus bradycardia	1 (0.5)	2 (0.5)	1.00
Related to the study drug	0 (0)	0 (0)	
Atrial fibrillation	1 (0.5)	3 (0.7)	1.00
Related to the study drug	0 (0)	0 (0)	
Atrio-ventricular block (syncope)	1 (0.5)	0 (0)	0.318
Related to the study drug	0 (0)	0 (0)	
Ischemic stroke	0 (0)	1 (0.2)	1.00
Related to the study drug	0 (0)	0 (0)	

Supplementary Table 11. Complete list of serious treatment-emergent adverse events (TEAE) Classified by System (continued)

	Placebo (n=189)	Anakinra (n=405)	P-value
Type of serious TEAE, n (%)			
Metabolic and electrolytes			
Hyperglycemia	2 (1.1)	1 (0.2)	0.239
Related to the study drug	0 (0)	0 (0)	
Hypoglycemia	1 (0.5)	2 (0.5)	1.00
Related to the study drug	0 (0)	0 (0)	1.00
Hypernatremia	1 (0.5)	4 (1.0)	1.00
Related to the study drug	1 (0.5)	0 (0)	1.00
Hyponatremia	0 (0)	2 (0.5)	1.00
Related to the study drug	0 (0)	0 (0)	
Hyperkalemia	2 (1.1)	0 (0)	0.101
Related to the study drug	1 (0.5)	0 (0)	
Hypocalcemia	0 (0)	1 (0.2)	1.00
Related to the study drug	0 (0)	0 (0)	
Blood and lymphatic tissue			
INR increase	1 (0.5)	1 (0.2)	0.535
Related to the study drug	0 (0)	0 (0)	
Prolongation of aPTT	1 (0.5)	0 (0)	0.318
Related to the study drug	0 (0)	0 (0)	
Increase of LFTs	2 (1.1)	4 (1.0)	1.00
Related to the study drug	1 (0.5)	2 (0.5)	
Anemia	3 (1.6)	2 (0.5)	0.333
Related to the study drug	0 (0)	0 (0)	
Neutropenia	0 (0)	1 (0.2)	1.00
Related to the study drug	0 (0)	1 (0.2)	
Lymphopenia	0 (0)	3 (0.7)	0.555
Related to the study drug	0 (0)	1 (0.2)	
Thrombocytopenia	1 (0.5)	0 (0)	0.318
Related to the study drug	0 (0)	0 (0)	
Skin			
Subcutaneous emphysema	1 (0.5)	0 (0)	0.318
Related to the study drug	0 (0)	0 (0)	

INR: international normalized ratio; LFTs: liver function tests

Supplementary Table 12. Complete list of non-serious treatment-emergent adverse events (TEAE) Classified by System

	Placebo (N=189)	Anakinra (N=405)	P-value
At least an adverse event — no. (%)	156 (82.5)	335 (82.7)	1.00
Type of adverse event — no. (%)			
Blood and lymphatic tissue			
Leukopenia	5 (2.6)	14 (3.5)	0.803
Grade 1	4 (2.1)	12 (3.0)	0.786
Grade 2	0 (0.0)	1 (0.2)	1.00
Grade 3	1 (0.5)	1 (0.2)	1.00
Neutropenia	1 (0.5)	12 (3.0)	0.072
Grade 1	1 (0.5)	8 (2.0)	0.284
Grade 2	0 (0.0)	4 (1.0)	0.315
Anemia	37 (19.6)	58 (14.3)	<0.0001
Grade 1	32 (16.9)	52 (12.8)	0.317
Grade 2	2 (1.1)	6 (1.5)	1.00
Grade 3	3 (1.6)	0 (0.0)	0.032
Thrombocytopenia	4 (2.1)	9 (2.2)	1.00
Grade 1	2 (1.1)	6 (1.5)	1.00
Grade 2	1 (0.5)	2 (0.5)	1.00
Grade 3	1 (0.5)	1 (0.2)	1.00
Thrombocytosis	13 (6.9)	24 (5.9)	0.716
Grade 1	11 (5.8)	24 (5.9)	1.00
Grade 2	2 (1.1)	0 (0.0)	0.101
Skin and dermis			
Reaction at injection site	0 (0.0)	2 (0.5)	1.00
Grade 1	0 (0.0)	2 (0.5)	1.00
Rash at the injection site	3 (1.5)	15 (3.7)	0.203
Grade 1	2 (1.1)	11 (2.7)	0.243
Grade 2	1 (0.5)	4 (1.0)	1.00
Gastrointestinal tract and liver			
Nausea, vomiting	1 (0.5)	9 (2.2)	0.181
Grade 1	0 (0.0)	8 (2.0)	0.061
Grade 2	1 (0.5)	1 (0.2)	1.00
Constipation	16 (8.5)	39 (9.6)	0.761
Grade 1	15 (7.9)	35 (8.6)	0.874
Grade 2	1 (0.5)	2 (0.5)	1.00
Grade 3	0 (0.0)	2 (0.5)	1.00
Diarrhea	8 (4.2)	14 (3.5)	0.645
Grade 1	7 (3.7)	13 (3.2)	0.808
Grade 2	1 (0.5)	1 (0.2)	1.00
Increase of liver function tests	63 (33.3)	145 (35.8)	0.580
Grade 1	48 (25.4)	111 (27.4)	0.432
Grade 2	11 (5.8)	24 (5.9)	1.00
Grade 3	4 (2.1)	10 (2.5)	1.00

Supplementary Table 12 Complete list of non-serious treatment-emergent adverse events (TEAE) Classified by System (continued)

Cardiovascular			
Bradycardia	19 (10.1)	36 (8.9)	0.880
Grade 1	15 (7.9)	31 (7.7)	1.00
Grade 2	3 (1.6)	4 (1.0)	1.00
Grade 3	1 (0.5)	1 (0.2)	1.00
Central nervous system			
Headache	8 (4.2)	16 (4.0)	1.00
Grade 1	7 (3.7)	13 (3.2)	0.808
Grade 2	1 (0.5)	1 (0.2)	1.00
Grade 3	0 (0.0)	1 (0.2)	1.00
Anxiety	11 (5.8)	33 (8.2)	0.400
Grade 1	8 (4.2)	22 (5.4)	0.688
Grade 2	3 (1.6)	11 (2.7)	0.564
Delirium	2 (1.1)	3 (0.7)	1.00
Grade 1	1 (0.5)	0 (0.0)	0.318
Grade 2	0 (0.0)	2 (0.5)	1.00
Grade 3	1 (0.5)	1 (0.2)	1.00
Creatinine increase	9 (4.8)	17 (4.2)	0.823
Grade 1	4 (2.1)	17 (4.2)	0.240
Grade 2	3 (1.6)	0 (0.0)	0.032
Grade 3	2 (1.1)	0 (0.0)	0.101
Metabolic and electrolytes			
Hyperglycemia	76 (40.2)	148 (36.5)	0.413
Grade 1	61 (32.3)	114 (28.1)	0.334
Grade 2	9 (4.8)	19 (4.7)	1.00
Grade 3	6 (3.2)	15 (3.7)	0.816
Hyponatremia	23 (12.2)	32 (7.9)	0.097
Grade 1	22 (11.6)	28 (6.9)	0.058
Grade 2	1 (0.5)	3 (0.7)	1.00
Grade 3	0 (0.0)	1 (0.2)	1.00
Hypernatremia	17 (9.0)	46 (11.4)	0.474
Grade 1	14 (7.4)	31 (7.7)	1.00
Grade 2	2 (1.1)	9 (2.2)	0.516
Grade 3	1 (0.5)	6 (1.5)	0.440
Hypokalemia	12 (6.3)	11 (2.7)	0.040
Grade 1	11 (5.8)	9 (2.2)	0.029
Grade 2	1 (0.5)	2 (0.5)	1.00
Hyperkalemia	13 (6.9)	36 (8.9)	0.522
Grade 1	7 (3.7)	21 (5.2)	0.535
Grade 2	5 (2.6)	10 (2.0)	1.00
Grade 3	1 (0.5)	5 (1.2)	0.670
Hypercalcemia	1 (0.5)	4 (1.0)	1.00
Grade 1	1 (0.5)	3 (0.6)	1.00
Grade 2	0 (0.0)	1 (0.2)	1.00
Hypocalcemia	20 (10.6)	32 (7.9)	0.279
Grade 1	14 (7.4)	19 (4.7)	0.183
Grade 2	6 (3.2)	11 (2.7)	0.793
Grade 3	0 (0.0)	2 (0.5)	1.00
Hypermagnesemia	1 (0.5)	2 (0.5)	1.00
Grade 1	1 (0.5)	2 (0.5)	1.00
Hypomagnesemia	1 (0.5)	3 (0.7)	1.00
Grade 1	1 (0.5)	3 (0.7)	1.00

Supplementary Table 13 Univariate and multivariate ordinal regression analysis of the WHO-CPS on day 28.
Comparisons between anakinra and placebo are done by univariate and multivariate ordinal regression analyses. Co-variates entered in the multivariate model were a) those used for stratified randomization according to the received advice by the COVID-ETF of the EMA; b) intake of remdesivir; and c) baseline values of IL-6, ferritin and respiratory ration above the median. The exact P-value of the comparison of anakinra vs placebo of the multivariate analyses is 2.3 $x10^{-7}$.

Variable	Univariate analysis			Multivariate analysis		
	Odds ratio	95% CIs	P-value	Odds ratio	95% CIs	P-value
Group of treatment (Anakinra vs placebo)	0.36	0.26-0.49	<0.0001	0.42	0.30-0.58	<0.0001
Intake of dexamethasone (Yes/No)	1.90	1.28-2.83	0.002	1.87	0.69-5.06	0.212
Severe COVID-19 by WHO (Yes/No)	1.95	1.31-2.90	0.001	0.94	0.35-2.52	0.906
BMI >30 kg/m^2 (Yes/No)	1.27	0.87-1.61	0.267	1.15	0.84-1.59	0.375
Country (Italy vs Greece)	1.18	0.74-1.88	0.482	1.58	0.93-2.72	0.093
Intake of remdesivir (Yes/No)	1.00	0.72-1.41	0.969	0.85	0.59-1.22	0.376
IL-6 >16.8 pg/ml (Yes/No)	1.56	1.16-2.11	0.004	1.62	1.19-2.22	0.002
Ferritin >585.2 ng/ml (Yes/No)	0.85	0.63-1.14	0.276	1.09	0.79-1.50	0.591
PaO_2/FiO_2 <237 (Yes/No)	2.10	1.56-2.84	<0.0001	1.79	1.28-2.52	0.001

BMI: body mass index; CI: confidence internal; FiO_2: fraction of inspired oxygen; IL: interleukin; PaO_2: partial arterial oxygen pressure; WHO: World Health Organization

Supplementary Table 14 Baseline absolute lymphocyte counts and concentrations of ferritin, IL-6 and suPAR among patients with low baseline CRP Patients with baseline CRP below the first quartile (i.e. 25.3 mg/l) were classified as low CRP.

	Lymphocytes (/mm^3), median (range)	Ferritin (ng/ml), median (range)	IL-6 (pg/ml), median (range)	suPAR (ng/ml), median (range)
Placebo (n= 46)	1015 (350-2350)	487.8 (29.9-2376.3)	8.8 (1.4-6263.0)	7.3 (6.1->15)
Anakinra (n= 100)	1020 (140-3300)	397.7 (58.4-6830.0)	11.1 (1.4-102.7)	7.3 (6.0->15)

CRP: C-reactive protein; IL: interleukin; suPAR: soluble urokinase plasminogen activator receptor

Supplementary Table 15 Univariate and multivariate ordinal regression analysis of the WHO-CPS on day 28 among patients with low CRP. Comparisons between anakinra and placebo are done by univariate and multivariate ordinal regression analyses. Co-variates entered in the multivariate model were those used for stratified randomization according to the received advice by the COVID-ETF of the EMA.

**Variable**	**Univariate analysis**			**Multivariate analysis**		
	**Odds ratio**	**95% CIs**	**P-value**	**Odds ratio**	**95% CIs**	**P-value**
Group of treatment (Anakinra vs placebo)	0.37	0.19-0.72	0.003	0.36	0.18-0.71	0.003
Intake of dexamethasone (Yes/No)	1.66	0.74-3.69	0.214	1.21	0.25-5.80	0.814
Severe COVID-19 by WHO (Yes/No)	1.99	0.90-4.40	0.089	1.59	0.34-7.48	0.558
BMI >30 kg/m^2 (Yes/No)	0.69	0.37-1.33	0.275	0.67	0.35-1.29	0.232
Country (Italy vs Greece)	1.81	0.69-4.69	0.222	2.57	0.92-7.11	0.069

BMI: body mass index; CI: confidence internal; FiO_2: fraction of inspired oxygen; IL: interleukin; PaO_2: partial arterial oxygen pressure; WHO: World Health Organization

Supplementary Table 16 Univariate and multivariate ordinal regression analysis of the WHO-CPS on day 28 among patients with COVID-associated hyperinflammatory syndrome (cHIS). cHIS is defined by the present of 2 or more of a set of six criteria introduced by Webb et al[7]*. This analysis involves 487 patients: 150 patients allocated to the placebo arm; and 337 patients allocated to the anakinra arm. Comparisons between anakinra and placebo are done by univariate and multivariate ordinal regression analyses. Co-variates entered in the multivariate model were those used for stratified randomization according to the received advice by the COVID-ETF of the EMA. The exact P-value of the comparison of anakinra vs placebo of the multivariate analyses is 7.8 x 10^{-7}.

**Variable**	**Univariate analysis**			**Multivariate analysis**		
	**Odds ratio**	**95% CIs**	**P-value**	**Odds ratio**	**95% CIs**	**P-value**
Group of treatment (Anakinra vs placebo)	0.39	0.28-0.57	<0.0001	0.41	0.28-0.58	<0.0001
Intake of dexamethasone (Yes/No)	1.99	1.28-3.09	0.002	1.59	0.58-4.38	0.367
Severe COVID-19 by WHO (Yes/No)	2.05	1.31-3.20	0.002	1.32	0.47-3.65	0.597
BMI >30 kg/m^2 (Yes/No)	1.21	0.86-1.67	0.273	1.11	0.79-1.57	0.538
Country (Italy vs Greece)	1.44	0.85-2.43	0.165	1.56	0.91-2.64	0.104

*Criteria are fever (defined as core temperature more than 38^0C); macrophage activation (defined as ferritin concentration 700 ng/ml or more); hematological dysfunction (defined as neutrophil/lymphocyte ratio 10 of more or a combination of hemoglobin 9.2 g/dl of less and absolute platelet count 100,000/mm^3 or less); coagulopathy (defined as D-dimer concentration 1.5 mg/l or more); hepatic injury (defined as lactate dehydrogenase concentration 400 U/l or more or aspartate aminotransferase 100 U/l or more); and cytokinemia (defined as interleukin-6 concentration 15 pg/ml or more or triglyceride concentration 150 mg/dl or more; or C-reactive protein concentration 150 mg/l or more).
BMI: body mass index; CI: confidence internal; WHO: World Health Organization

Supplementary Table 17 Univariate and multivariate ordinal regression analysis of the WHO-CPS on day 28 among patients positive according to the predictive criteria for progression into with COVID-associated cytokine storm. This analysis involves 210 patients: 64 patients allocated to the placebo arm; and 146 patients allocated to the anakinra arm. The criteria are introduced by Caricchio et al[8]*. Comparisons between anakinra and placebo are done by univariate and multivariate ordinal regression analyses. Co-variates entered in the multivariate model were those used for stratified randomization according to the received advice by the COVID-ETF of the EMA.

**Variable**	**Univariate analysis**			**Multivariate analysis**		
	**Odds ratio**	**95% CIs**	**P-value**	**Odds ratio**	**95% CIs**	**P-value**
Group of treatment (Anakinra vs placebo)	0.36	0.20-0.62	<0.0001	0.39	0.22-0.66	0.001
Intake of dexamethasone (Yes/No)	3.63	1.51-8.70	0.004	3.20	0.29-35.19	0.346
Severe COVID-19 by WHO (Yes/No)	3.46	1.37-8.74	0.009	1.07	0.08-13.62	0.958
BMI >30 kg/m^2 (Yes/No)	1.69	1.02-2.82	0.043	1.54	0.92-2.57	0.100
Country (Italy vs Greece)	1.21	0.52-2.87	0.649	1.00	0.42-2.38	0.993

*The predictive criteria for cytokine storm are mandatory and variables from each of three clusters. Mandatory criteria that should all be met are signs/symptoms of COVID-19, molecular confirmation of SARS-CoV-2, radiological ground-glass opacities, ferritin more than 250 ng/ml and C-reactive protein more 46mg/l. The first cluster comprises three variables one of which should be met (albumin less than 2.8 g/dl; lymphocytes less than 10.2%; and absolute neutrophil count more than 11400/mm^3). The second cluster comprises five variables one of which should be met (alanine aminotransferase more than 60 U/l; aspartate aminotransferase more than 87 U/l; D-dimers more than 4.93 mg/l; lactate dehydrogenase more than 416 U/l; and troponin I more than 1.09 ng/ml).
The third cluster comprises four variables one of which should be met (anion gap less than 6.8 mmol/l; chloride more than 106 mmol/l; potassium more than 4.9/mm^3; and blood urea nitrogen/creatinine ratio more than 29).
BMI: body mass index; CI: confidence internal; WHO: World Health Organization

Supplementary Table 18 Incidence of severe respiratory failure and/or death until day 14 for patients scoring for at least two of AST, CRP, ferritin and NLR above the defined cut-offs*. Analysis involved all patients for which all four variables were available at baseline before start of the study drug. Comparisons between anakinra and placebo are done by the Fisher' s exact test.

	Placebo, n (%)	Anakinra, n (%)	P-value
	None or only one of CRP >50 mg/l, NLR> 5.5, ferritin >700 ng/ml or AST >44 U/l		
No severe respiratory failure/death	60 (90.9)	145 (87.9)	0.647
Severe respiratory failure/death	6 (9.1)	20 (12.1)	
Total (n)	66	165	
	At least two CRP >50 mg/l, NLR> 5.5, ferritin >700 ng/ml or AST >44 U/l		
No severe respiratory failure/death	61 (55.5)	155 (72.8)	0.003
Severe respiratory failure/death	49 (44.5)	58 (27.2)	
Total (n)	110	213	

*The derivation of the cut-offs is provided at Extended Data Figure 8.
AST: aspartate aminotransferase; CRP: C-reactive protein; NLR: neutrophil/lymphocyte ratio

Supplementary Table 19 Incidence of severe respiratory failure and/or death until day 28 according to risk as this is defined by AST, CRP, ferritin and NLR before start of the study drug separately for patients allocated to the placebo group and for patients allocated to the Anakinra group. Analysis involved all patients for which all four variables were available at baseline before start of the study drug. Comparisons between patients without and with risk as defined by the biomarkers are done by the Fisher' s exact test. The comparisons between the odds ratios (ORs) are done by the Tarone's test and by the Breslow-Day test.

	**≤1 of CRP >50 mg/l, NLR> 5.5, ferritin >700 ng/ml, AST >44 U/l**	**≥2 of CRP >50 mg/l, NLR> 5.5, ferritin >700 ng/ml, AST >44 U/l**	**OR (95%CI)**
	Placebo, n (%)		
No severe respiratory failure/death	60 (90.9)	59 (53.6)	8.64
Severe respiratory failure/death	6 (9.1)	51 (46.4)	3.45-21.67
Total (n)	66	110	
	Anakinra, n (%)		
No severe respiratory failure/death	144 (87.3)	154 (72.9)	2.63*
Severe respiratory failure/death	21 (12.7)	59 (27.7)	1.52-4.54
Total (n)	165	213	

*P: 0.026 between the two ORs by both the Tarone's test and the Breslow-Day test.
AST: aspartate aminotransferase; CRP: C-reactive protein; NLR: neutrophil/lymphocyte ratio
