# Supplementary material for: Early treatment of COVID-19 with anakinra guided by soluble urokinase plasminogen receptor plasma levels: a double-blind, randomized controlled phase 3 trial
Source: Nat Med. 2021 Sep 3;27(10):1752–60. doi: 10.1038/s41591-021-01499-z (PMC8516650; doi:10.1038/s41591-021-01499-z)
Supplement: Supplementary file 2 — Reporting Summary [file 41591_2021_1499_MOESM2_ESM.pdf]

nature research

Corresponding author(s): Evangelos J. Giamarellos-Bourboulis

Last updated by author(s): Jul 16, 2021

Reporting Summary

Nature Research wishes to improve the reproducibility of the work that we publish. This form provides structure for consistency and transparency in reporting. For further information on Nature Research policies, see our Editorial Policies and the Editorial Policy Checklist.

Statistics

For all statistical analyses, confirm that the following items are present in the figure legend, table legend, main text, or Methods section.

n/a	Confirmed	
☐	☒	The exact sample size (n) for each experimental group/condition, given as a discrete number and unit of measurement
☐	☒	A statement on whether measurements were taken from distinct samples or whether the same sample was measured repeatedly
☐	☒	The statistical test(s) used AND whether they are one- or two-sided<br>*Only common tests should be described solely by name; describe more complex techniques in the Methods section.*
☐	☒	A description of all covariates tested
☐	☒	A description of any assumptions or corrections, such as tests of normality and adjustment for multiple comparisons
☐	☒	A full description of the statistical parameters including central tendency (e.g. means) or other basic estimates (e.g. regression coefficient) AND variation (e.g. standard deviation) or associated estimates of uncertainty (e.g. confidence intervals)
☐	☒	For null hypothesis testing, the test statistic (e.g. F, t, r) with confidence intervals, effect sizes, degrees of freedom and P value noted<br>*Give P values as exact values whenever suitable.*
☒	☐	For Bayesian analysis, information on the choice of priors and Markov chain Monte Carlo settings
☒	☐	For hierarchical and complex designs, identification of the appropriate level for tests and full reporting of outcomes
☐	☒	Estimates of effect sizes (e.g. Cohen's d, Pearson's r), indicating how they were calculated

Our web collection on statistics for biologists contains articles on many of the points above.

Software and code

Policy information about availability of computer code

Data collection	http://www.save-more.org/
Data analysis	SPSS v. 26.0

For manuscripts utilizing custom algorithms or software that are central to the research but not yet described in published literature, software must be made available to editors and reviewers. We strongly encourage code deposition in a community repository (e.g. GitHub). See the Nature Research guidelines for submitting code & software for further information.

Data

Policy information about availability of data

All manuscripts must include a data availability statement. This statement should provide the following information, where applicable:

- Accession codes, unique identifiers, or web links for publicly available datasets
- A list of figures that have associated raw data
- A description of any restrictions on data availability

Requests for deidentified patient data by researchers with proposed use of the data can be made to corresponding author with specific data needs, analysis plans and dissemination plans. Those requests will be reviewed by a study steering committee and the study sponsor for release upon publication. Contact: egiamarel@med.uoa.gr

Field-specific reporting

Please select the one below that is the best fit for your research. If you are not sure, read the appropriate sections before making your selection.

☒ Life sciences ☐ Behavioural & social sciences ☐ Ecological, evolutionary & environmental sciences

For a reference copy of the document with all sections, see nature.com/documents/nr-reporting-summary-flat.pdf

Life sciences study design

All studies must disclose on these points even when the disclosure is negative.

Sample size

The sample size was calculated based on the finding from the phase II SAVE trial that 42% of comparators and 16.3% of anakinra-treated patients by day 28 were presented with 6 or more points of the WHO-CPS. To achieve such a difference in the WHO-CPS scores with 90% power at the 5% level of significance, allocation of 200 patients to SoC and placebo treatment and 400 patients to SoC and anakinra treatment were planned.

Data exclusions

12 patients withdrew consent and requested removal of all data, leaving a final ITT analysis cohort of 594 patients; 189 patients were allocated to the SoC and placebo arm, and 405 patients were allocated to the SoC and anakinra arm. Only one patient was lost to follow-up

Replication

The analysis of the primary endpoint should have been supported by three analyses: comparison of the WHO-CPS by day 14; logistic regression analysis separately for patients at the two spectra of WHO-CPS at day 28; and time progression to respiratory failure by day 14. The first spectrum of the WHO-CPS was defined as patients fully recovered with negative viral load (WHO-CPS 0 points) contrary to patients with persistent disease (WHO-CPS between points 1 to 10). The second spectrum was defined as patients pointed 6 or more in the WHO-CPS (severe hospitalized and dead) contrary to patients pointed 5 or less. Five sensitivity analyses were conducted to assess robustness: exclusion of population deviating from the SoC; population receiving at least 7 doses of the study drug; complete analysis set; responder analysis treating missing values as non-responders; and comparison of the unadjusted and the adjusted treatment effects. The three confirmatory analyses fully supported the clinical benefit of anakinra treatment.

Randomization

Patients with suPAR 6 ng/ml or more were electronically 1:2 randomized into treatment with placebo or anakinra using four randomization strata: classification into moderate or severe disease using the WHO definition; need for dexamethasone intake; body mass index (BMI) more than 30 kg/m2; and country. Study drug was prepared by an unblinded pharmacist with access to the electronic study system using a separate username and a password.

Blinding

Patients and treating physicians were blind to allocation arm. Study drug was prepared by an unblinded pharmacist with access to the electronic study system using a separate username and a password. Study drug administration was done by a blind study nurse.

Reporting for specific materials, systems and methods

We require information from authors about some types of materials, experimental systems and methods used in many studies. Here, indicate whether each material, system or method listed is relevant to your study. If you are not sure if a list item applies to your research, read the appropriate section before selecting a response.

Materials & experimental systems

n/a	Involved in the study	
☒	☐	Antibodies
☒	☐	Eukaryotic cell lines
☒	☐	Palaeontology and archaeology
☒	☐	Animals and other organisms
☐	☒	Human research participants
☐	☒	Clinical data
☒	☐	Dual use research of concern

Methods

n/a	Involved in the study	
☒	☐	ChIP-seq
☒	☐	Flow cytometry
☒	☐	MRI-based neuroimaging

Human research participants

Policy information about studies involving human research participants

Population characteristics

Enrolled patients were adults of either gender; with molecular diagnosis of infection by SARS-CoV-2; with involvement of the lower respiratory tract as confirmed by chest computed tomography or X-ray; in need for hospitalization; and with plasma suPAR 6 ng/ml or more. Main exclusion criteria were: ratio or partial oxygen pressure to fraction of inspired oxygen less than 150; need of non-invasive ventilation (CPAP or BPAP) or mechanical ventilation; neutropenia; stage IV malignancy; end-stage renal disease; severe hepatic failure; immunodeficiencies; and chronic intake of corticosteroids and biological anti-cytokine drugs.

Recruitment

All patients admitted to hospital with COVID-19 pneumonia providing written informed consent were screened for inclusion

Recruitment	and exclusion criteria.
Ethics oversight	The protocol was approved by the National Ethics Committee of Greece (approval 161/20) and by the Ethics Committee of the National Institute for Infectious Diseases Lazzaro Spallanzani, IRCCS in Rome (01.02.2021) (EudraCT number, 2020-005828-11; ClinicalTrials.gov NCT04680949).

Note that full information on the approval of the study protocol must also be provided in the manuscript.

Clinical data

Policy information about clinical studies

All manuscripts should comply with the ICMJE guidelines for publication of clinical research and a completed CONSORT checklist must be included with all submissions.

Clinical trial registration	EudraCT number, 2020-005828-11; ClinicalTrials.gov NCT04680949
Study protocol	Available within submitted material
Data collection	SAVE-MORE is a prospective double-blind RCT conducted in 37 study sites (29 in Greece and 8 in Italy). From December 2020 through March 2021, 1060 patients were screened and 606 were randomized. 12 patients withdrew consent and requested removal of all data, leaving a final ITT analysis cohort of 594 patients; 189 patients were allocated to the SoC and placebo arm, and 405 patients were allocated to the SoC and anakinra arm. Only one patient was lost to follow-up. Data collection was made in an electronic CRF: http://www.save-more.org/
Outcomes	The primary study endpoint was the overall comparison of the distribution of frequencies of the scores from the 11-point WHO Clinical Progression ordinal Scale (CPS) between the two arms of treatment at Day 28. Secondary endpoints included the changes of WHO-CPS by days 14 and 28 from the baseline (before start of the study drug); the change of SOFA score by day 7 from baseline; the time until hospital discharge; the time of stay in the intensive care unit (ICU) for patients eventually admitted to the ICU; and the comparison of biomarkers. Results were expressed as the odds ratio (OR) and 95% confidence intervals (CI). The two basic assumptions of the model, i.e., proportional odds and the goodness-of-fit test were checked. According to EMA's COVID-ETF advice the variables used for stratified randomization entered as co-variates in the multivariate model, i.e., disease severity, intake of dexamethasone, BMI more than 30, and country. According to the same advice, the analysis of the primary endpoint should have been supported by three analyses: comparison of the WHO-CPS by day 14; logistic regression analysis separately for patients at the two spectra of WHO-CPS at day 28; and time progression to respiratory failure by day 14. The first spectrum of the WHO-CPS was defined as patients fully recovered with negative viral load (WHO-CPS 0 points) contrary to patients with persistent disease (WHO-CPS between points 1 to 10). The second spectrum was defined as patients pointed 6 or more in the WHO-CPS (severe hospitalized and dead) contrary to patients pointed 5 or less. Five sensitivity analyses were conducted to assess robustness: exclusion of population deviating from the SoC; population receiving at least 7 doses of the study drug; complete analysis set; responder analysis treating missing values as non-responders; and comparison of the unadjusted and the adjusted treatment effects. Analysis was conducted using IBM SPSS Statistics v. 26.0. All P values were two-sided and any P value <0.05 was considered as statistically significant.
