# Supplementary figures and images for: Early treatment of COVID-19 with anakinra guided by soluble urokinase plasminogen receptor plasma levels: a double-blind, randomized controlled phase 3 trial
Source: Nat Med. 2021 Sep 3;27(10):1752–60. doi: 10.1038/s41591-021-01499-z (PMC8516650; doi:10.1038/s41591-021-01499-z)

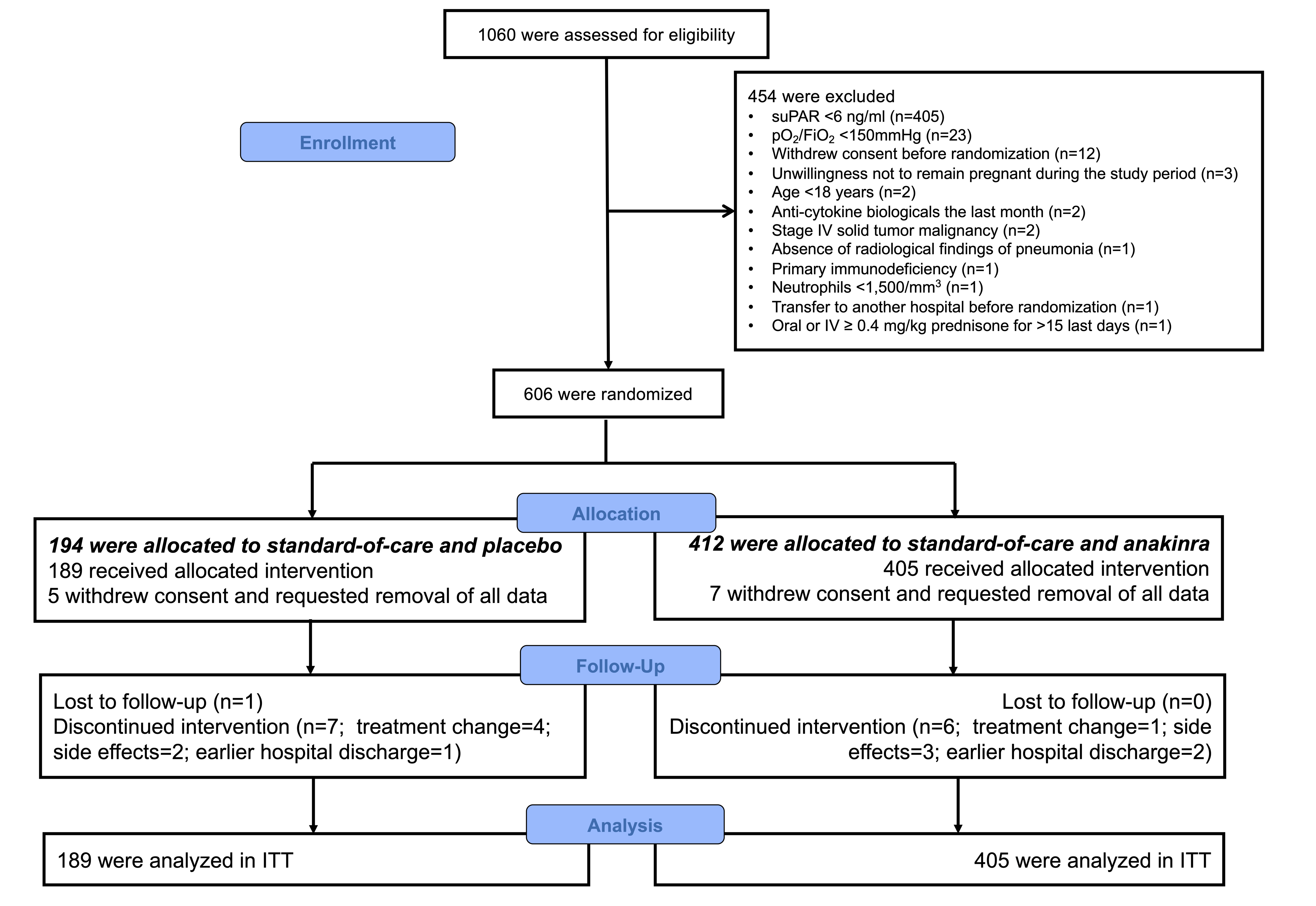

Supplement: Supplementary file 3 — Source Data Fig. 1 [file 41591_2021_1499_MOESM3_ESM.tiff]

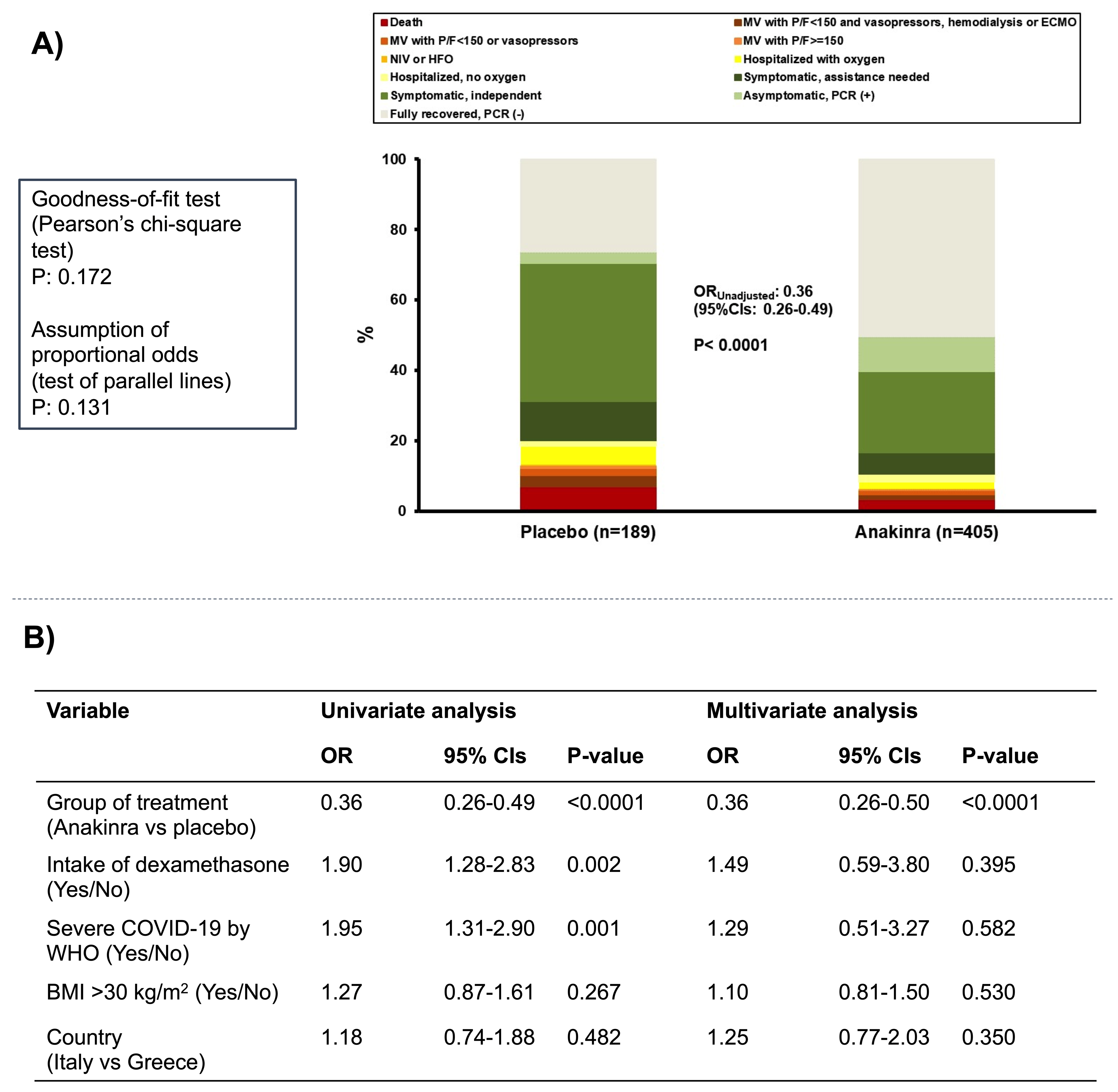

Supplement: Supplementary file 4 — Source Data Fig. 2 [file 41591_2021_1499_MOESM4_ESM.tiff]

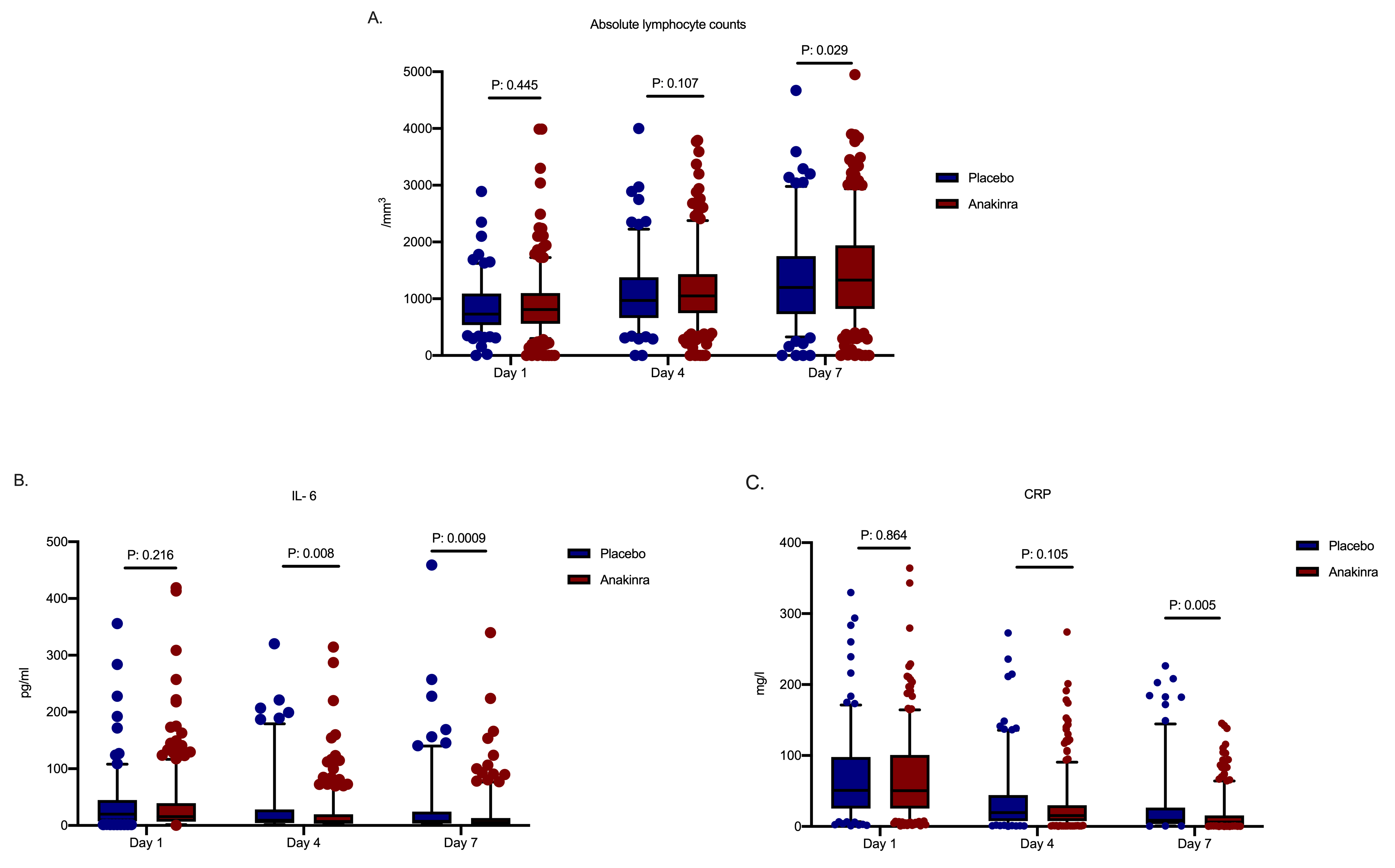

Supplement: Supplementary file 5 — Source Data Fig. 3 [file 41591_2021_1499_MOESM5_ESM.tiff]

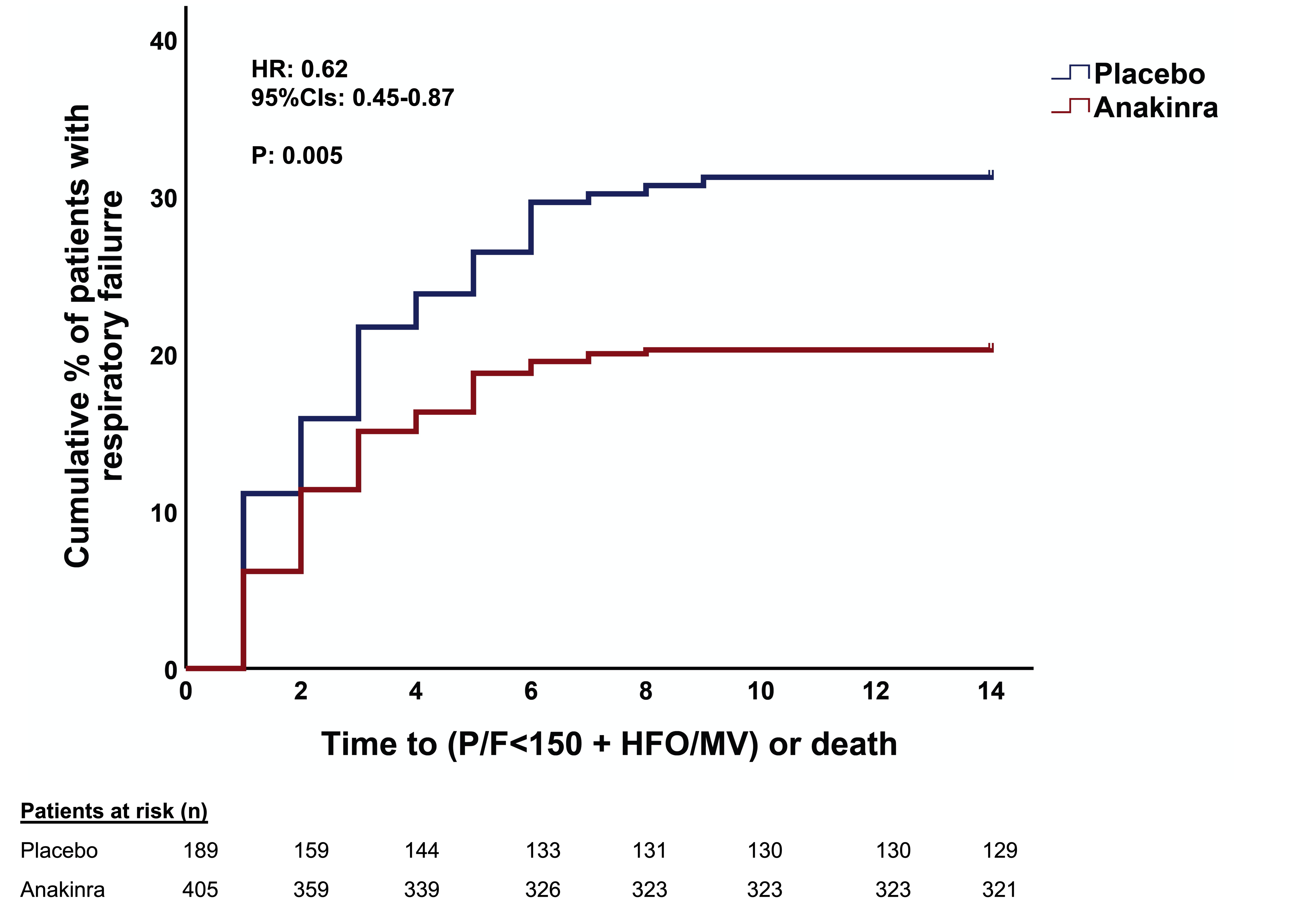

Supplement: Supplementary file 7 — Source Data Extended Data Fig. 2 [file 41591_2021_1499_MOESM7_ESM.tiff]

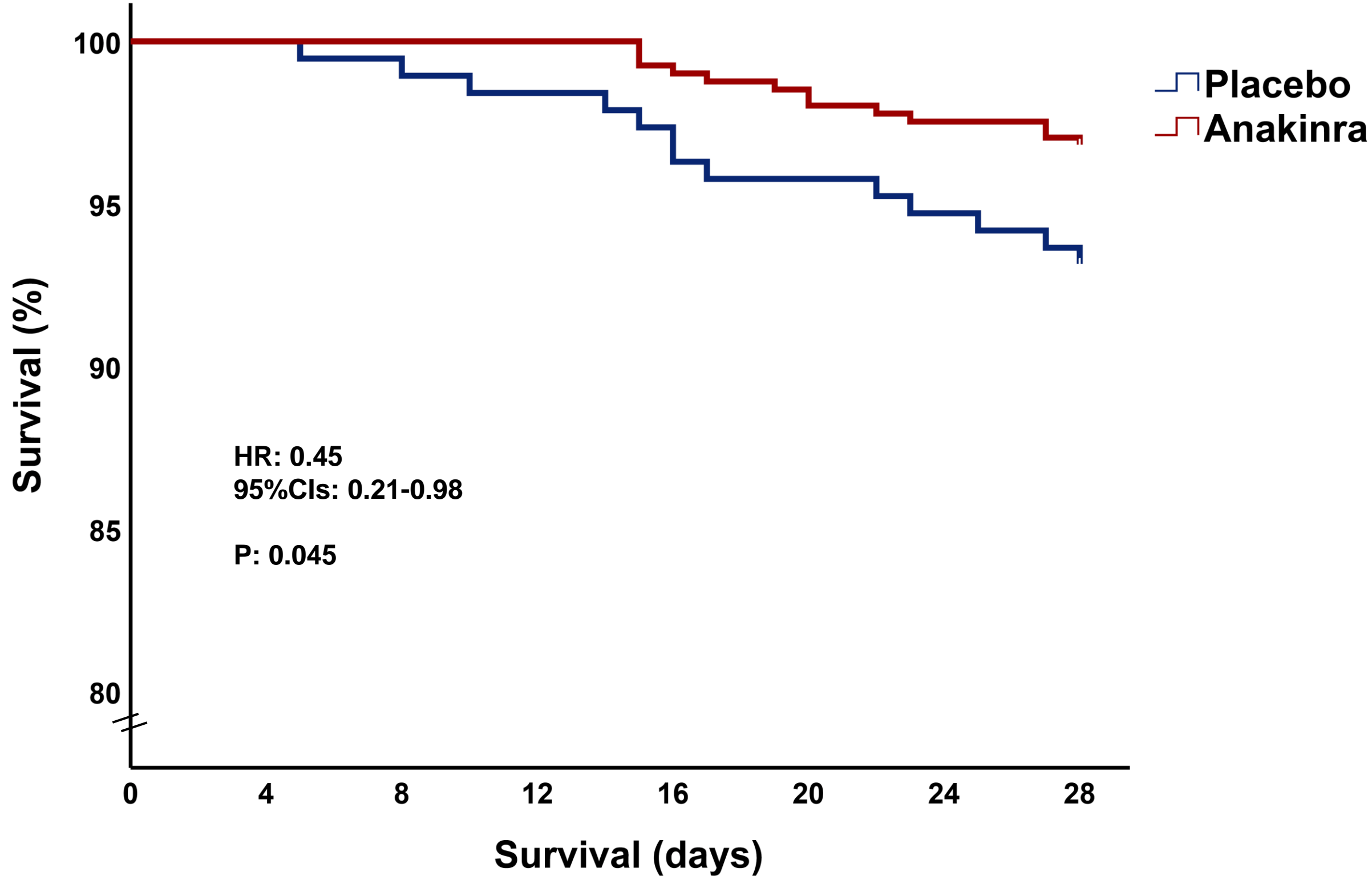

**Patients at risk (n)**

|          |     |     |     |     |     |     |     |     |
|----------|-----|-----|-----|-----|-----|-----|-----|-----|
| Placebo  | 189 | 189 | 187 | 186 | 182 | 181 | 179 | 176 |
| Anakinra | 405 | 405 | 405 | 405 | 401 | 397 | 395 | 392 |

Supplement: Supplementary file 8 — Source Data Extended Data Fig. 3 [file 41591_2021_1499_MOESM8_ESM.pdf]

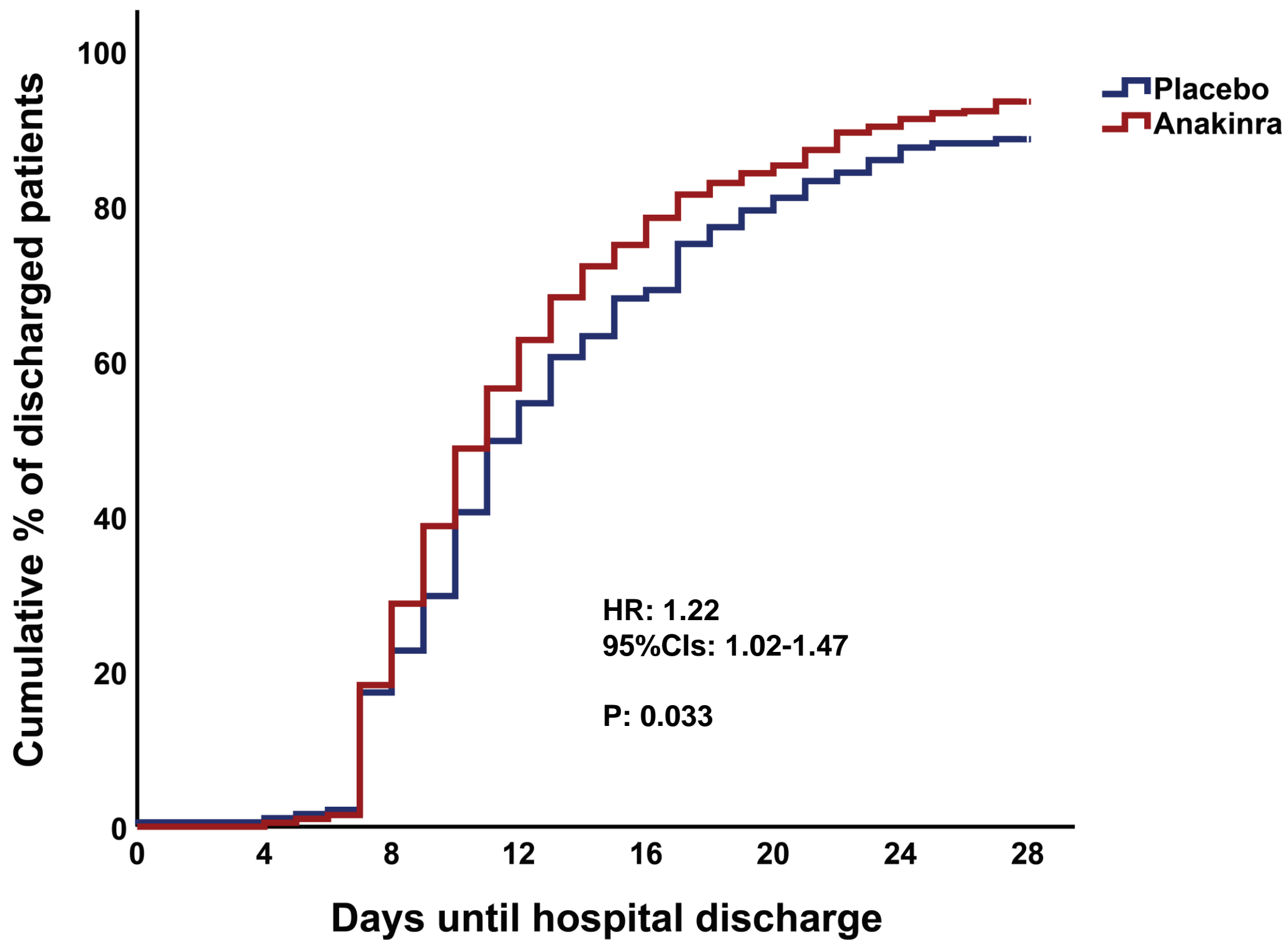

**Patients at risk (n)**

|          |     |     |     |     |    |    |    |    |
|----------|-----|-----|-----|-----|----|----|----|----|
| Placebo  | 189 | 187 | 137 | 88  | 61 | 39 | 27 | 20 |
| Anakinra | 405 | 403 | 290 | 154 | 91 | 64 | 48 | 24 |

Supplement: Supplementary file 9 — Source Data Extended Data Fig. 4 [file 41591_2021_1499_MOESM9_ESM.pdf]

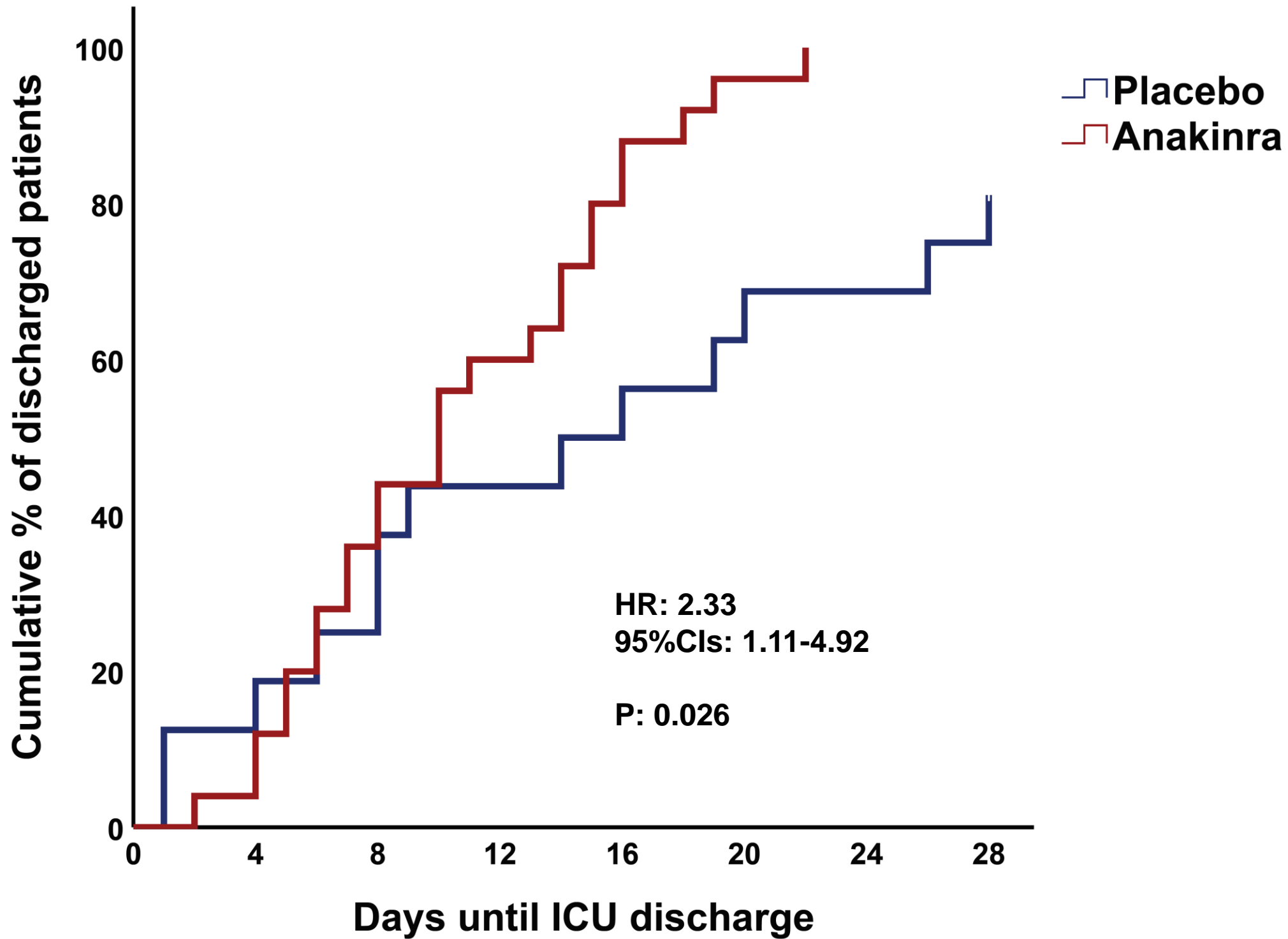

**Patients at risk (n)**

|          |    |    |    |    |   |   |   |   |
|----------|----|----|----|----|---|---|---|---|
| Placebo  | 16 | 13 | 10 | 9  | 7 | 5 | 5 | 4 |
| Anakinra | 26 | 22 | 14 | 10 | 3 | 1 | 0 | 0 |

Supplement: Supplementary file 10 — Source Data Extended Data Fig. 5 [file 41591_2021_1499_MOESM10_ESM.pdf]

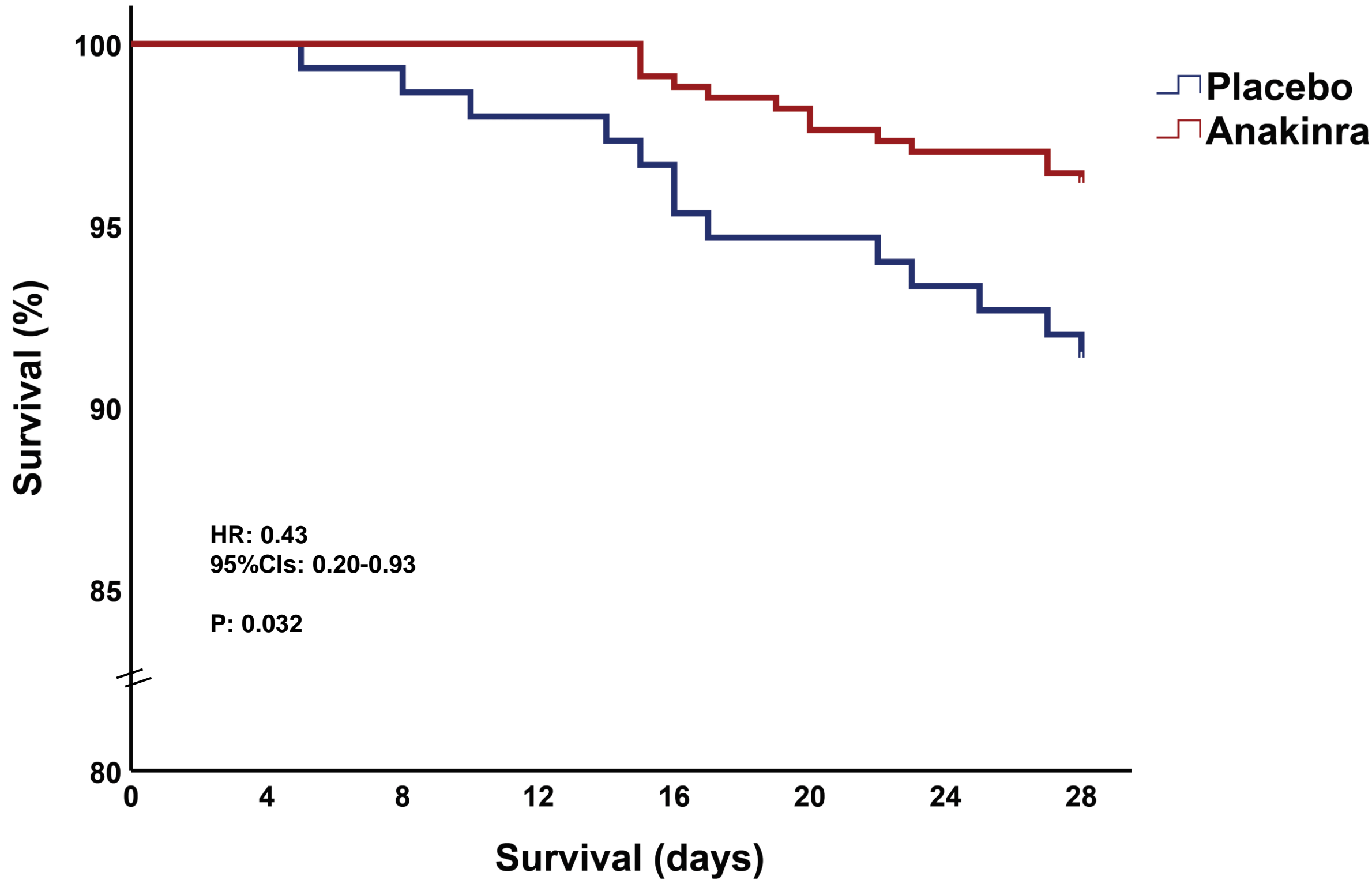

**Patients at risk (n)**

|          |     |     |     |     |     |     |     |     |
|----------|-----|-----|-----|-----|-----|-----|-----|-----|
| Placebo  | 150 | 150 | 149 | 147 | 143 | 142 | 140 | 137 |
| Anakinra | 337 | 337 | 337 | 337 | 333 | 329 | 327 | 324 |

Supplement: Supplementary file 11 — Source Data Extended Data Fig. 6 [file 41591_2021_1499_MOESM11_ESM.pdf]

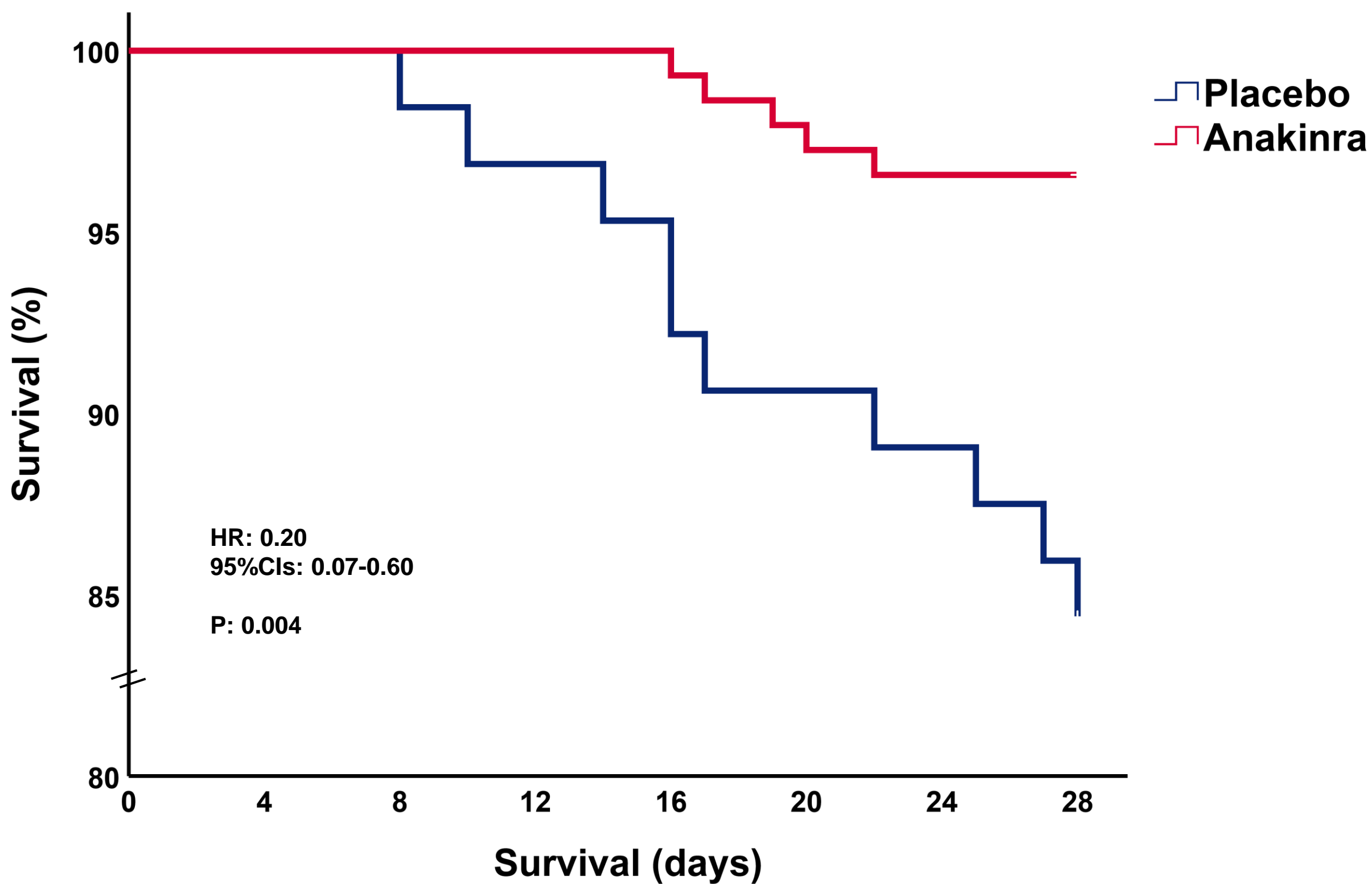

**Patients at risk (n)**

|          |     |     |     |     |     |     |     |     |
|----------|-----|-----|-----|-----|-----|-----|-----|-----|
| Placebo  | 64  | 64  | 63  | 62  | 60  | 59  | 58  | 54  |
| Anakinra | 146 | 146 | 146 | 146 | 145 | 142 | 141 | 141 |

Supplement: Supplementary file 12 — Source Data Extended Data Fig. 7 [file 41591_2021_1499_MOESM12_ESM.pdf]
