# Supplementary material for: Early treatment of COVID-19 with anakinra guided by soluble urokinase plasminogen receptor plasma levels: a double-blind, randomized controlled phase 3 trial
Source: Nat Med. 2021 Sep 3;27(10):1752–60. doi: 10.1038/s41591-021-01499-z (PMC8516650; doi:10.1038/s41591-021-01499-z)

Goodness-of-fit test
(Pearson’s chi-square test)
P: 0.128

Assumption of proportional odds
(test of parallel lines)
P: 0.078

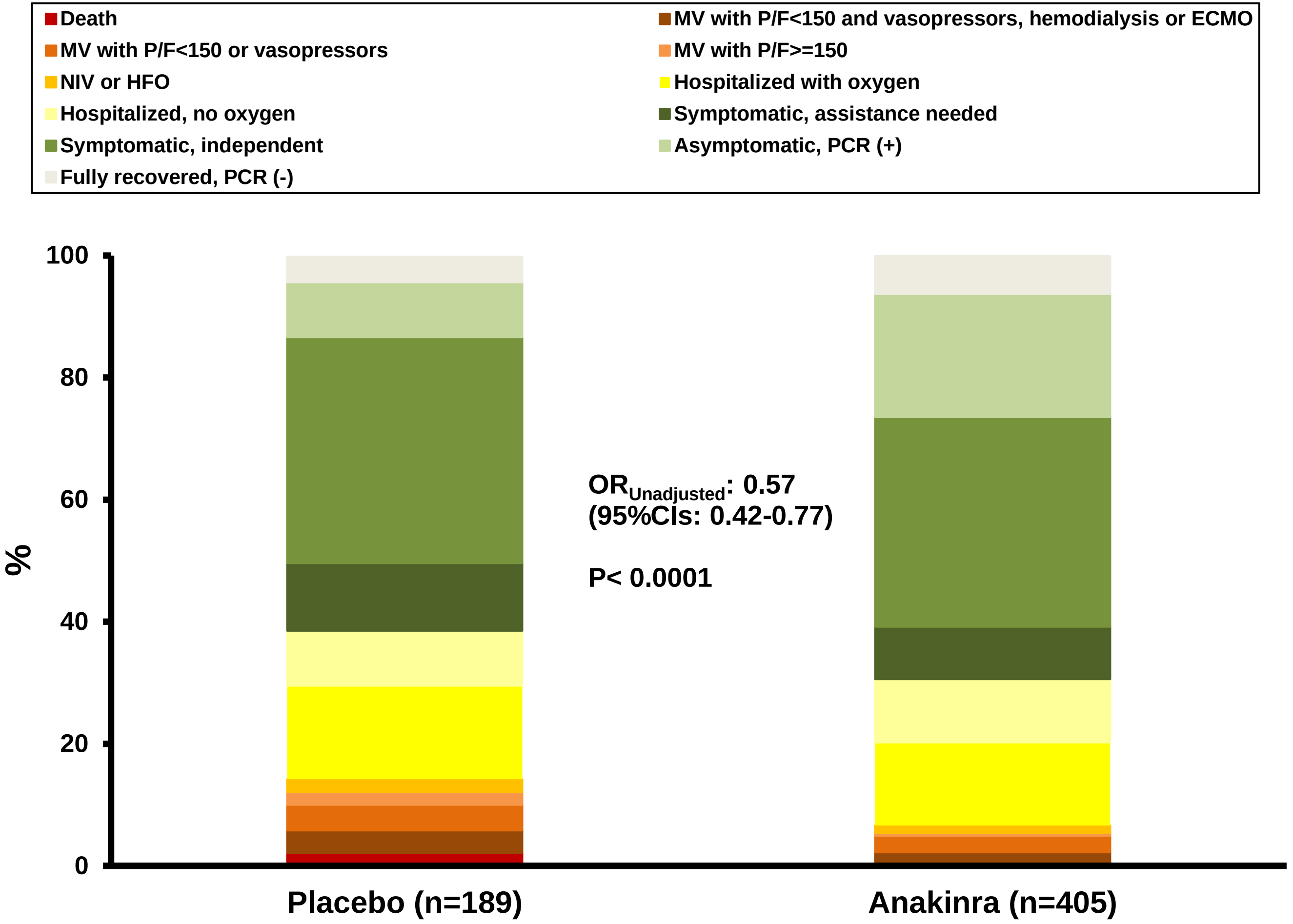

Supplement: Supplementary file 6 — Source Data Extended Data Fig. 1 [file 41591_2021_1499_MOESM6_ESM.pdf]
