# Supplementary material for: Early treatment of COVID-19 with anakinra guided by soluble urokinase plasminogen receptor plasma levels: a double-blind, randomized controlled phase 3 trial
Source: Nat Med. 2021 Sep 3;27(10):1752–60. doi: 10.1038/s41591-021-01499-z (PMC8516650; doi:10.1038/s41591-021-01499-z)
Supplement: Supplementary file 13 — Source Data Extended Data Fig. 8 [file 41591_2021_1499_MOESM13_ESM.pdf]

Aspartate aminotransferase (AST)

A)

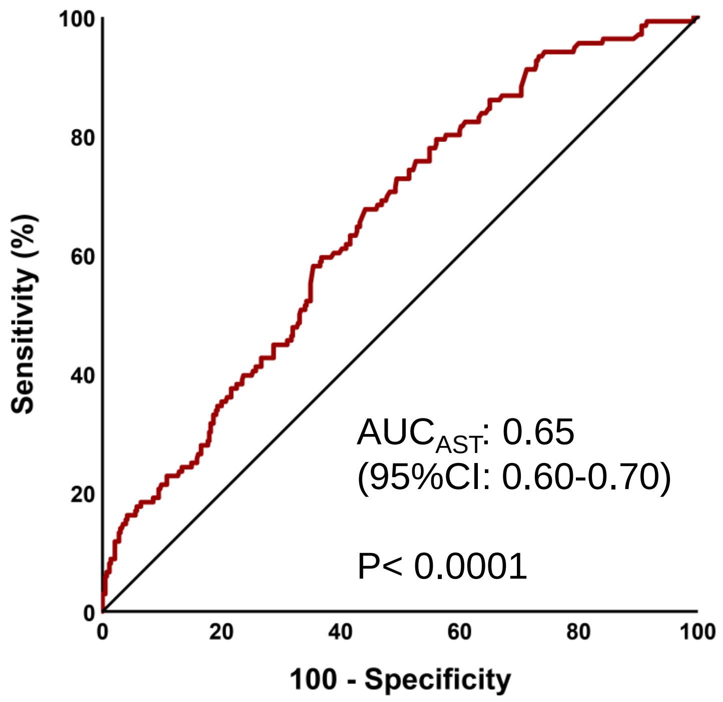

B)

	Severe respiratory failure/Death (+) (n)	Severe respiratory failure/Death (-) (n)	Total
AST >44 U/l	73<br>Sensitivity= 54.9%<br>PPV= 33.3%	146	219
AST ≤ 44 U/l	60	275<br>Specificity= 65.3%<br>NPV= 82.1%	335
Total (n)	133	421	554

OR: 2.29 (95%CI: 1.54-3.40); P< 0.0001

C-reactive protein (CRP)

E)

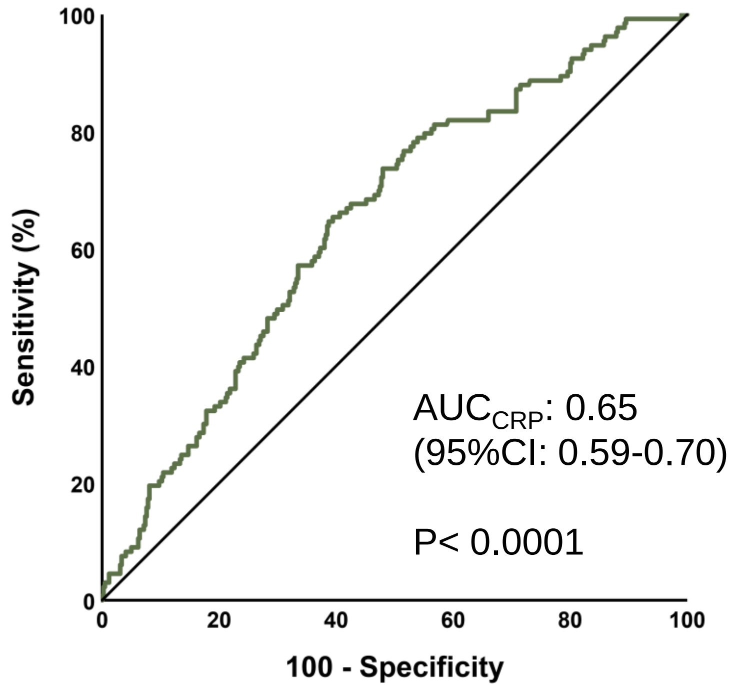

F)

	Severe respiratory failure/Death (+) (n)	Severe respiratory failure/Death (-) (n)	Total
CRP >50 mg/l	91<br>Sensitivity= 68.4%<br>PPV= 32.3%	191	282
CRP ≤ 50 mg/l	42	230<br>Specificity= 54.6%<br>NPV= 84.6%	272
Total (n)	133	421	554

OR: 2.61 (95%CI: 1.73-3.94); P< 0.0001

Neutrophil/lymphocyte ratio (NLR)

C)

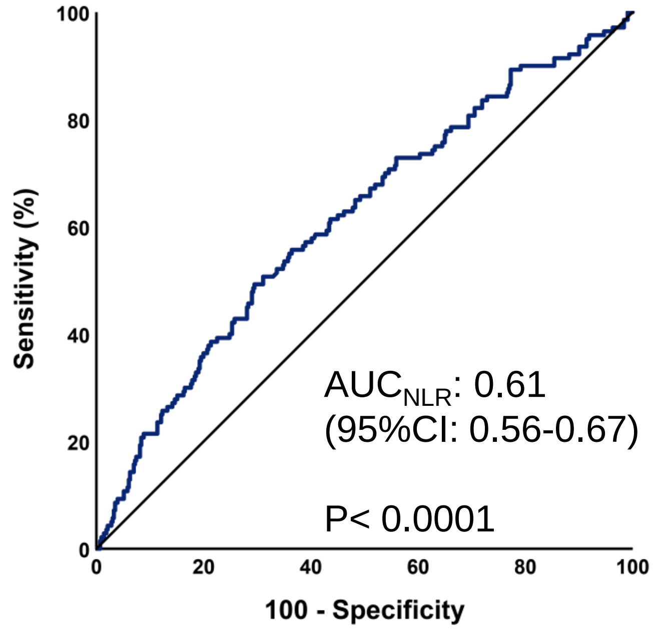

D)

	Severe respiratory failure/Death (+) (n)	Severe respiratory failure/Death (-) (n)	Total
NLR >5.5	85<br>Sensitivity= 63.7%<br>PPV= 30.1%	197	272
NLR ≤ 5.5	48	224<br>Specificity= 53.2%<br>NPV= 82.4%	282
Total (n)	133	421	554

OR: 2.01 (95%CI: 1.35-3.01); P: 0.001

Ferritin

G)

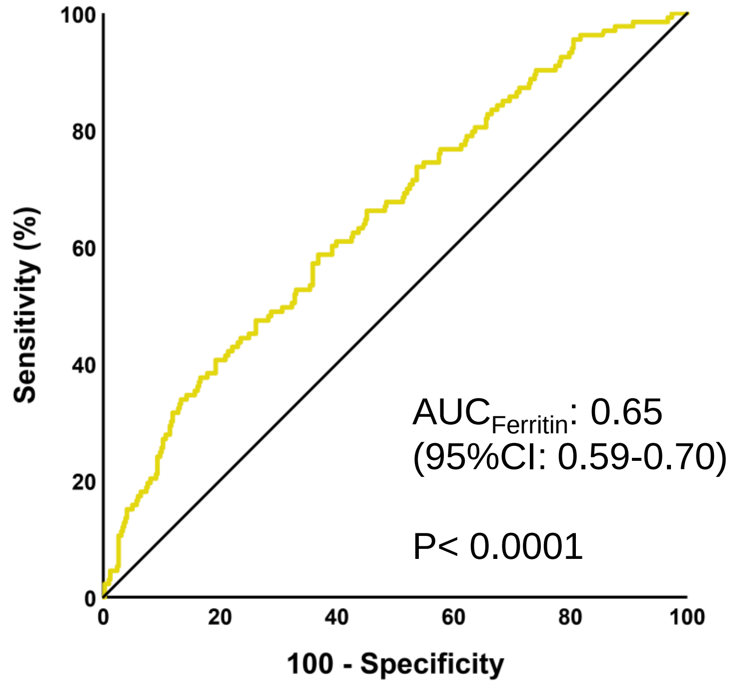

H)

	Severe respiratory failure/Death (+) (n)	Severe respiratory failure/Death (-) (n)	Total
Ferritin >700 ng/ml	78<br>Sensitivity= 58.6%<br>PPV= 32.4%	163	241
Ferritin ≤ 700 ng/ml	55	258<br>Specificity= 61.3%<br>NPV= 82.4%	313
Total (n)	133	421	554

OR: 2.25 (95%CI: 1.51-3.33); P< 0.0001
